# Oligosaccharides from the seeds of *Dolichos lablab L.* promote gut microbial metabolite L-arginine production to alleviate cyclophosphamide-induced immunosuppression

**DOI:** 10.3389/fimmu.2025.1587426

**Published:** 2025-07-16

**Authors:** Ben Liu, Jiayi Jin, Yuming Zhou, Zhipeng Shang, Peng Meng, Maoru Du, Feng Geng, Xue Gao, Feng Zhao, Zhenguo Su, Xiaohong Pan

**Affiliations:** ^1^ School of Pharmacy, Binzhou Medical University, Yantai, China; ^2^ Department of Laboratory Medicine, Yantai Affiliated Hospital of Binzhou Medical University, Yantai, China; ^3^ The Key Laboratory of Prescription Effect and Clinical Evaluation of State Administration of Traditional Chinese Medicine of China, Yantai, China; ^4^ Yantai Hospital of Traditional Chinese Medicine, Binzhou Medical University, Yantai, China; ^5^ Binzhou Medical University, Yantai Hospital of Traditional Chinese Medicine, Yantai, China

**Keywords:** *Dolichos lablab* L., oligosaccharide, gut microbiome, prebiotics, immunosuppression, chemotherapy

## Abstract

**Introduction:**

*Dolichos lablab L*. is a nutritionally and medicinally significant legume, yet research on its bioactive oligosaccharides remains limited. This study investigates the potential of *Dolichos lablab L*. oligosaccharides to ameliorate cyclophosphamide (CTX)-induced immunosuppression and intestinal damage.

**Methods:**

Crude oligosaccharides were purified to yield mixture SRSV (86% sugar content), comprising sucrose, raffinose, stachyose, and verbascose (mass ratio 19:16.8:50:14.2). Immunosuppressed mice (CTX-induced) were treated with SRSV (150 mg/kg). Gut microbiota (GM) diversity was analyzed via 16S rRNA sequencing, and serum metabolites were profiled using metabolomics. GM-depleted mice (antibiotic-treated) and L-arginine supplementation experiments were used for mechanistic validation.

**Results:**

SRSV preserved intestinal villi integrity, reversed CTX-induced immune organ atrophy, and restored the CD4+T/CD8+T ratio. It enhanced bone marrow hematopoiesis, elevated peripheral white blood cell and lymphocyte counts, and modulated serum TNF-α levels. SRSV increased GM diversity, enriching beneficial taxa (e.g., *Ruminococcus, UBA1819, Anaerofustis*) while reducing pathogenic Atopobiaceae. Antibiotic-induced GM depletion abrogated SRSV’s protective effects. Metabolomics identified L-arginine as a key upregulated metabolite, linked to arginine biosynthesis. L-arginine supplementation alone replicated SRSV’s immunoprotective outcomes.

**Discussion:**

SRSV attenuates CTX-induced immunosuppression through GM-dependent mechanisms and L-arginine-mediated immunomodulation. GM integrity is essential for SRSV efficacy, as its depletion abolishes protection. The restitution of L-arginine levels underpins SRSV’s capacity to restore immune homeostasis.

**Conclusion:**

SRSV from *Dolichos lablab L*. is a promising natural adjuvant for mitigating chemotherapy-induced immunosuppression and intestinal injury, acting via GM modulation and arginine biosynthesis pathways.

## 1 Introduction

Globally, the incidence and mortality rates of cancer are escalating, positioning it as a leading cause of mortality and a substantial barrier to extending human longevity (1, 2). Chemotherapy, a prevalent treatment for tumors, functions primarily by inhibiting the rapid proliferation of malignant cells. However, cells such as hair follicles, immune, bone marrow, and epithelial cells, which share rapid proliferative properties, are also indiscriminately targeted and destroyed during chemotherapy (3). This indiscriminate cellular damage can precipitate severe adverse effects, such as immunosuppression, gastrointestinal mucosal injuries, and disturbances in the GM (4–7). These adverse effects frequently necessitate the premature discontinuation of chemotherapy, reduce its efficacy, and facilitate tumor metastasis, consequently diminishing patient survival. Therefore, the development of effective strategies to mitigate these adverse effects is imperative to enhance the therapeutic outcomes of chemotherapy.

Prebiotics have been shown to enhance immunity, and oligosaccharides, the most extensively studied form of prebiotics, exert beneficial effects on host health by influencing the GM. These oligosaccharides are resistant to gastrointestinal digestion and absorption, but they provide a carbon source for specific probiotics. They can be fermented by bacteria in the gut, participating in the regulation of the microenvironment and promoting gut health (8, 9). During the early stages of life, oligosaccharides from breast milk are pivotal in shaping the neonatal GM and modulating metabolic product profiles (10), thereby safeguarding infants against colitis and bolstering resistance to viral infections (11, 12). Bletilla oligosaccharides (BO), derived from the traditional Chinese medicinal herb Bletilla striata, have been shown to ameliorate glucose intolerance and insulin resistance through modulation of the GM and intestinal metabolites, while also effectively attenuating chronic inflammation and preserving intestinal barrier integrity (13). Low-molecular-weight Lycium barbarum oligosaccharides (LBO) demonstrate superior antioxidant activity and gastrointestinal digestibility compared to high-molecular-weight Lycium barbarum polysaccharides *in vitro*. Supplementation with LBO has been shown to restore the structural equilibrium of gut bacterial communities, promote the proliferation of beneficial bacteria such as *Bacillus*, *Tyzzerella*, *Fournierella*, and *Coriobacteriaceae UCG-002* in the gastrointestinal tract, and ameliorate changes in microbial metabolism. Furthermore, LBO reduces serum levels of inflammatory cytokines and hepatic hydroxyproline, enhances mitochondrial function in both the gut and liver, and alleviates hepatic fibrosis in mice (14). β-Manno-oligosaccharides (β-MOS) are selectively fermented by intestinal microbiota, promoting the growth of beneficial bacteria and the production of metabolites, such as short-chain fatty acids (15, 16). Numerous studies on functional oligosaccharides have demonstrated that they can maintain human health by regulating intestinal flora, reducing intestinal inflammation, enhancing immunity, and through many other mechanisms.

Traditional Chinese herbs are one of the primary sources of functional oligosaccharides. *Dolichos lablab L.* has both high food value and high medicinal value and has been used as nutritious food and herbal medicine for thousands of years. The polysaccharides and oligosaccharides derived from *Dolichos lablab L.* are key bioactive components of the plant. Previous studies have shown that *Dolichos lablab L.* polysaccharides play a role in regulating GM composition and treating diseases. Li Wenjuan et al. purified a non-starch polysaccharide, named WHBP, from mature *Dolichos lablab L.* seeds. They found that WHBP restored intestinal barrier function by modulating GM composition and exhibited hypoglycemic effects in type 2 diabetic rats, contributing to diabetes management (17). Chen Lei et al. extracted crude polysaccharides (DSP and DFP) and ethanol extracts (DSE and DFE) from *Dolichos lablab L.* seeds (DS) and flowers (DF), respectively. Their study demonstrated that DSE and DFE suppressed oxidative stress, reduced the production of inflammatory factors, and preserved intestinal barrier integrity in mice with ulcerative colitis. 16S rRNA gene sequencing and metabolomics analyses reveal that both polysaccharides and ethanol extracts alleviate ulcerative colitis by modulating the GM structure and reversing dysregulated metabolism in the host (18). Therefore, it is evident that *Dolichos lablab L.* polysaccharides exhibit effects such as modulating GM, anti-inflammatory properties, and protecting the intestinal barrier. However, whether *Dolichos lablab L.* oligosaccharides exert similar effects has not been reported. Research on *Dolichos lablab L.* oligosaccharides is currently limited, likely due to the challenges in their isolation and purification, which have impeded further investigation. It has been demonstrated that converting polysaccharides into oligosaccharides enhances their biological activity and more effectively promotes the growth of probiotics, such as *Bifidobacteria* and *Lactobacilli (* 19).

In this study, a crude oligosaccharide extract derived from the seeds of *Dolichos lablab L.* was purified to obtain the oligosaccharide mixture SRSV. It is hypothesized that SRSV can alleviate chemotherapy-induced immunosuppression and intestinal damage. The underlying mechanism may involve enhancing immunity and protecting intestinal barrier integrity by modulating the GM and their metabolites. The findings will provide novel strategies for mitigating chemotherapy side effects and enhancing the efficacy of cancer treatment, while offering new insights into the pharmacological effects and bioactive components of *Dolichos lablab L*.

## 2 Materials and methods

### 2.1 The source of materials

The crude *Dolichos lablab L.* oligosaccharide was provided by Sichuan Weikeqi Biological Technology CO (Sichuan, China). The standard oligosaccharides including sucrose, raffinose, stachyose, and verbascose were purchased from Alading Reagent Co. (Shanghai, China). Cellobiose and T series dextrans were purchased from Yuanye Biotechnology (Shanghai, China). Sodium hydroxide solution (12.5 mol/L) for HPAEC analysis was purchased from Sigma-Aldrich (St. Louis, MO, USA).

CTX was purchased from Baxter Oncology GmbH (Halle, Germany). The levels of tumor necrosis factor-α (TNF-α) in the mice serum were tested using the commercial ELISA kits based on submitted instructions (Shanghai Enzyme-linked Biotechnology Co., Ltd, Shanghai, China). PE anti-mouse CD3 and FITC anti-mouse CD8a were purchased from BioLegend (San Diego, USA). APC anti-mouse CD4 was purchased from Thermo Fisher Scientific (Waltham, MA, USA). Ampicillin, kanamycin, streptomycin, and vancomycin were purchased from Melone Pharmaceutical Co., Ltd. (Dalian, China).

### 2.2 Preparation of the SRSV

#### 2.2.1 Preparation and purification of SRSV

The crude oligosaccharide from *Dolichos lablab L.* was applied to a DEAE-52 (JNC corporation, Tokyo, Japan) anion exchange column (40 cm × 2.5 cm), and the sequential elution was carried out with deionized water, 0.17, 0.34, 0.51, 0.68, and 0.85 mol/L NaCl aqueous solution. The elution curve was derived from the total carbohydrate amount in each tube quantified by the phenol–sulfuric acid method with glucose as a standard (20). A major fraction (DLO-W) eluted by deionized water was obtained and subjected to further purification and bioassays.

The DLO-W was analyzed on a Waters high-performance gel permeation chromatography (HPGPC) equipped with a Shodex OHpak SB-806M HQ column as previously described (21). A standard curve (lgMw = -1.3994T+14.973, R2 = 0.995) was established using cellobiose and the T series dextrans (T-1, T-2, T-3, T-5, and T-20) to evaluate the average molecular weight.

To further confirm the oligosaccharide structures in *Dolichos lablab L.*, DLO-W was loaded on a gel filtration column (120 cm × 2 cm) over Sephadex LH-20 (Pharmacia Biotech AB, Uppsala, Sweden), with a stepwise gradient of EtOH−H2O (from 20% to 0%, v/v) at a flow rate of 0.4 mL/min. The DLO-W, eluates, and standard oligosaccharides were analyzed by a high-performance anion exchange chromatography (HPAEC) coupled with a CarboPac™ PA100 column and a pulsed amperometric detector (Thermo Fisher Scientific, USA). Elution was performed with 0.2 mol/L NaOH for preliminary analysis and with 0.05 mol/L NaOH for complete separation at a flow rate of 0.7 mL/min.

#### 2.2.2 NMR spectroscopic analysis

24 mg of stachyose was dissolved in 0.5 mL D_2_O for analysis. The NMR spectra including ^1^H, ^13^C, HSQC, and HMBC spectra were measured with a Bruker Avance III HD 600 NMR spectrometer (600 MHz for ^1^H and 150 MHz for ^13^C, respectively).

### 2.3 Construction and treatment of immunosuppressed mouse models

ICR mice (5-6 weeks old, 22 ± 2 g, male) used in this study were obtained from the Pengyue Experimental Animal Center (No. SCXK20190003, Jinan, China). The animals were housed in a specific pathogen-free (SPF) facility with a 12-hour light/dark cycle, maintained at approximately 25°C and 40%–70% humidity. Mice had ad libitum access to food and water. After a 7-day acclimatization period, the experiments were conducted. The experimental protocols adhered to the ARRIVE guidelines, and all animal treatments and experiments complied with the National Guidelines for the Ethical Review of Laboratory Animal Welfare of the People’s Republic of China (GB/T 35892-2018), as approved by the Animal Ethics Committee of Binzhou Medical University (No. 2015005).

Healthy ICR mice were randomly assigned to five groups (n = 6 per group): normal control (CTR), immunosuppressed model (CTX), low-dose SRSV (SRSV-L), medium-dose SRSV (SRSV-M), and high-dose SRSV (SRSV-H). The CTR and CTX groups were gavaged with saline, while the SRSV-L, SRSV-M, and SRSV-H groups received SRSV solutions at concentrations of 75 mg/kg, 150 mg/kg, and 300 mg/kg, respectively, prepared in saline. The gavage volume for all groups was 10 mL/kg, administered once daily at a fixed time. After 14 days of gavage, the CTX, SRSV-L, SRSV-M, and SRSV-H groups were intraperitoneally injected with CTX at a dose of 100 mg/kg, while the CTR group was injected with saline. The injection volume for all groups was 10 mL/kg. Four days after CTX injection, mice were anesthetized, and samples were collected for analysis.

### 2.4 Establishment of the antibiotic-induced microbiota-depletion mouse model

Broad-spectrum antibiotics, including sodium ampicillin, kanamycin, and streptomycin (1 mg/mL each), as well as vancomycin (0.5 mg/mL), were administered to the mice via drinking water ad libitum. After seven days of treatment, fecal samples were collected and inoculated onto solid and liquid microbial culture media to evaluate microbial growth. After overnight incubation at 37°C, no colony formation was observed, confirming the successful establishment of the AIMD mouse model.

### 2.5 Sample collection

On the day before the conclusion of the experiment, mouse feces were collected in sterile cryogenic tubes and stored at −80°C for subsequent analysis. Mouse blood was collected, and centrifuged at 4°C at 3000 rpm for 10 minutes, and the serum was separated and stored at −80°C. The spleen and thymus were rinsed with cold, sterile saline, blotted dry with filter paper, and weighed. Mouse femur tissue was isolated, while the distal colon and ileum were collected and fixed in 4% paraformaldehyde for 48 hours. The remaining portions of the colon, ileum, and their contents were stored at −80°C.

### 2.6 Calculation of immune organ index

At the end of the experiment, the body weight of each group of mice was recorded prior to euthanasia. The thymus and spleen were carefully dissected, and the surrounding fascia and adipose tissue were meticulously removed. The organs were then weighed, and the organ index was calculated using the formula: Organ weight (g) × 100/Body weight (g).

### 2.7 Cytokines detection by ELISA

The TNF-α level in mouse serum was measured using an ELISA kit (Shanghai Enzyme-linked Biotechnology Co., Ltd., Shanghai, China) following the manufacturer’s instructions. After the reaction was terminated, absorbance at 450 nm was measured using a microplate reader (Biotek, USA), and the serum cytokine concentration was calculated.

### 2.8 Flow cytometry

The fresh spleen was carefully separated from connective tissue and thoroughly homogenized. The cell concentration was adjusted to 1 × 10^6^ cells/mL, and the cell morphology was observed under a microscope. Red blood cell lysis buffer was added to remove erythrocytes. For flow cytometric analysis of T cell subsets, samples were first stained with CD3-PE to identify T lymphocytes. Subsequently, within the CD3^+^ T cell population, CD4-APC and CD8a-FITC were used to determine the proportions of CD4^+^ and CD8^+^ T cells, respectively. All staining procedures were conducted at 4°C for 30 minutes in the dark. After incubation, the samples were washed twice with PBS and analyzed using a BD Canto II flow cytometer (BD Biosciences, USA).

### 2.9 Histological analysis

Jejunum and femur tissues were fixed in 4% paraformaldehyde for 48 hours, followed by dehydration. The femur tissues were decalcified in a decalcification solution for 14 days before dehydration. The embedded tissues were sectioned into 4-μm thick slices, fixed onto slides, and stained with Hematoxylin and Eosin (HE). Histological differences between groups were observed using an optical microscope (OLYMPUS BX53, Japan), and images were captured. Intestinal sections were scanned using a Pannoramic MIDI scanner, and villus length was measured with CaseViewer 2.4.

### 2.10 Analysis of GM and metabolites

Fecal samples from mice were collected under sterile conditions, flash-frozen in liquid nitrogen, and stored at −80°C for later analysis. DNA was extracted from the fecal samples using the Omega Bio-tek kit (Norcross, GA, USA) for assessing GM diversity and composition. PCR amplification, 16S rRNA sequencing, metabolite sample preparation, and LC-MS analysis were performed at Majorbio Biotech (Shanghai, China). The sequencing data were analyzed and processed using the Majorbio Cloud Platform (www.majorbio.com).

### 2.11 Statistical analysis

For comparisons between two groups, an unpaired, two-tailed Student’s t-test was used. To identify significantly altered bacterial populations, the Wilcoxon rank-sum test was performed using the Majorbio Cloud Platform, with p-values calculated using a two-tailed FDR-corrected method. A significance threshold of 0.05 was applied, and 95% confidence intervals were determined using the bootstrap algorithm. For comparisons involving more than two groups, one-way or two-way ANOVA followed by Dunnett’s test was applied. Data are presented as mean ± SD, with p < 0.05 considered statistically significant. All statistical analyses were performed using GraphPad Prism (GraphPad Software Inc., USA).

## 3 Results

### 3.1 Characterization and purification of oligosaccharides

#### 3.1.1 Purification and oligosaccharide profile analysis of DLO-W

**Figure 1:**
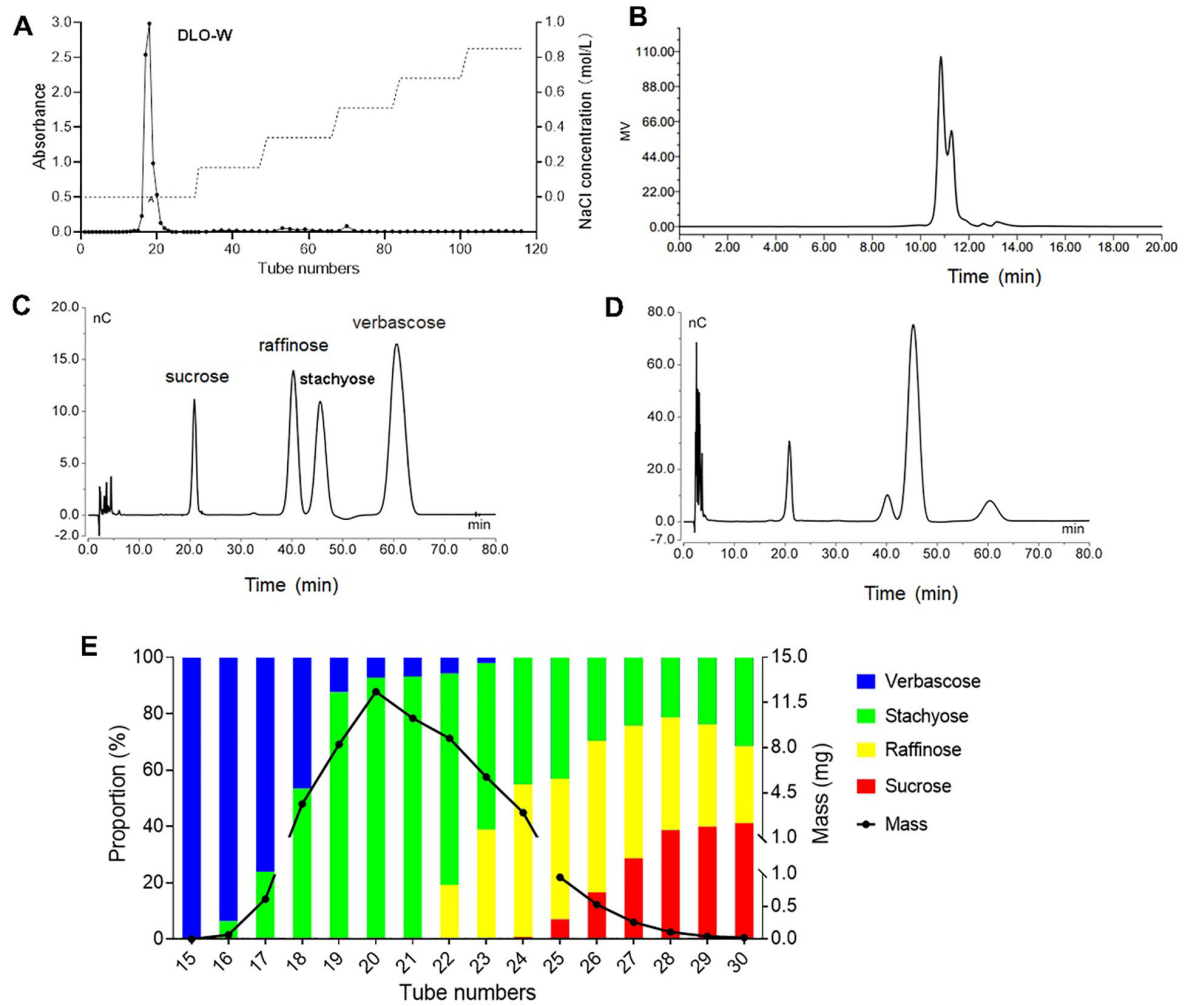
Purification and oligosaccharide profile analysis of DLO-W. **(A)** DEAE-cellulose elution curve of the crude oligosaccharide. **(B)** HPGPC chromatogram of DLO-W. HPAEC chromatograms for mixed oligosaccharide standards **(C)** and DLO-W **(D, E)** Sephadex LH-20 elution curve of DLO-W.

The crude *Dolichos lablab L.* oligosaccharide was fractionated using DEAE-cellulose-52 column chromatography, sequentially eluted with water, 0.17, 0.34, 0.51, 0.68, and 0.85 mol/L NaCl aqueous solutions. The major fraction, containing 92% of the total eluate mass, was obtained in the water elution and was designated as DLO-W (**Figure 1A**). Additionally, after purification by anion exchange chromatography, the total sugar content of the crude oligosaccharide increased from 44% to greater than 86% in DLO-W.

Analysis of carbohydrate compositions by HPGPC indicated that DLO-W consisted of oligosaccharides with a molecular weight of less than 1378 Da, with the major oligosaccharide having a molecular weight of approximately 630 Da (tR = 10.84 min) (**Figure 1B**). Next, HPAEC coupled with a CarboPac PA100 column was employed for oligosaccharide profile analysis. The chromatogram displayed four distinct absorption peaks typical of raffinose family oligosaccharides, which are widespread across the plant kingdom, particularly in the seeds of legumes (22). Further optimization of the chromatographic conditions resulted in the complete separation of the four oligosaccharides in DLO-W. As shown in **Figures 1C, D**, the four oligosaccharides were identified as sucrose, raffinose, stachyose, and verbascose, with a mass ratio of 19:16.8:50:14.2, confirming that stachyose is the major oligosaccharide, in agreement with the HPGPC results. These results indicate that DLO-W represents the total oligosaccharides of *Dolichos lablab L.* The purified DLO-W (with a purity greater than 86%) was used for subsequent biological activity studies and was renamed SRSV.

Moreover, DLO-W was further purified using Sephadex LH-20 column chromatography, and the oligosaccharides in each fraction were analyzed by HPAEC. As illustrated in **Figure 1E**, most of the stachyose was separated from DLO-W. Over 84% of the stachyose, with a purity of 87.9%, was obtained in four fractions, of which more than 68%, with a purity of 93.1%, was obtained in two fractions. However, the separation efficiency of Sephadex LH-20 for sucrose and raffinose was limited. The elution fractions were concentrated, lyophilized, and subjected to NMR analysis for structural confirmation.

#### 3.1.2 Characterization of stachyose

**Figure 2:**
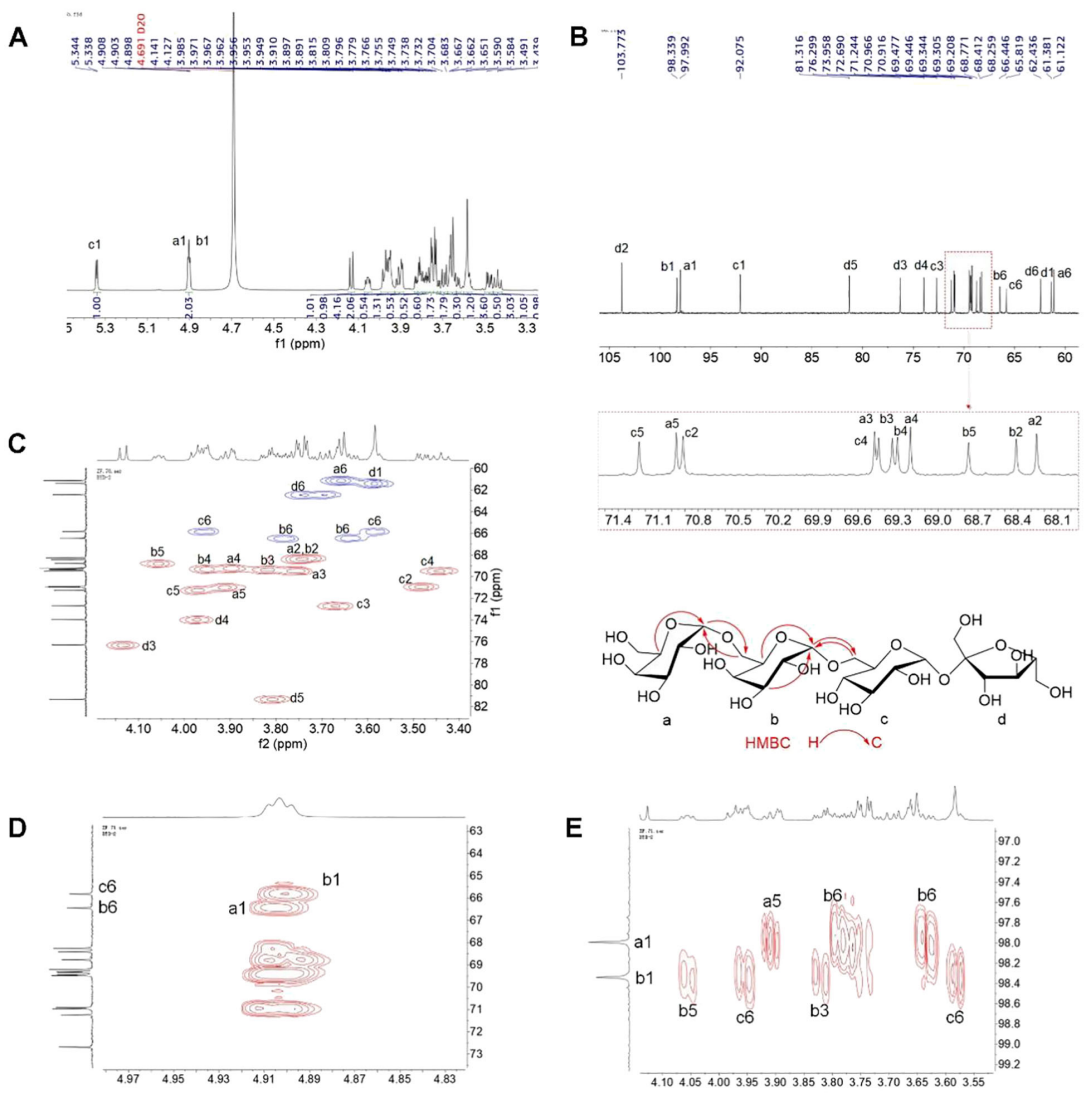
1D and 2D NMR spectrum of stachyose. **(A)** ^1^H NMR, **(B)** ^13^C NMR, **(C)** HSQC (red correlations: CH/CH_3_; blue correlations: CH_2_), and **(D, E)** HMBC.

The 1D and 2D NMR spectra of the major oligosaccharide are shown in **Figure 2**. The ^1^H and ^13^C NMR spectra displayed three sets of anomeric proton signals at δ (4.91, 4.90)/(97.99, 98.34), and 5.34/92.08 ppm, along with a significantly shielded quaternary carbon signal at δ 103.77 ppm, indicating the presence of four monosaccharide residues. The NMR spectra were identical to those of stachyose. The only minor differences were that the overlapped carbon signals reported in the reference were distinguishable at δ 70.97, 70.92, 69.48, 69.45, 69.34, and 69.31 (**Figures 2A, B**), and were assigned to C-a5, C-c2, C-c4, C-a3, C-b3, and C-b4, respectively, based on the associations observed in the HSQC spectrum (**Figure 2C**). The key HMBC correlations of CH-a1 with CH_2_-b6, and CH-b1 with CH_2_-b6, assigned the overlapped anomeric signals at δ 4.91/97.99 to the terminal galactose (a), and those at 4.90/98.34 to →6)-Gal-(1→ (b) (**Figures 2D, E**). Based on the comprehensive interpretation of the 2D NMR spectra and reference to the literature data (23), the full assignments of the NMR data were made and summarized in **Supplementary Table 1**. Similarly, the other three oligosaccharides were identified as sucrose, raffinose, and verbascose, respectively (data not shown).

### 3.2 SRSV effectively alleviates CTX-induced immune suppression

**Figure 3:**
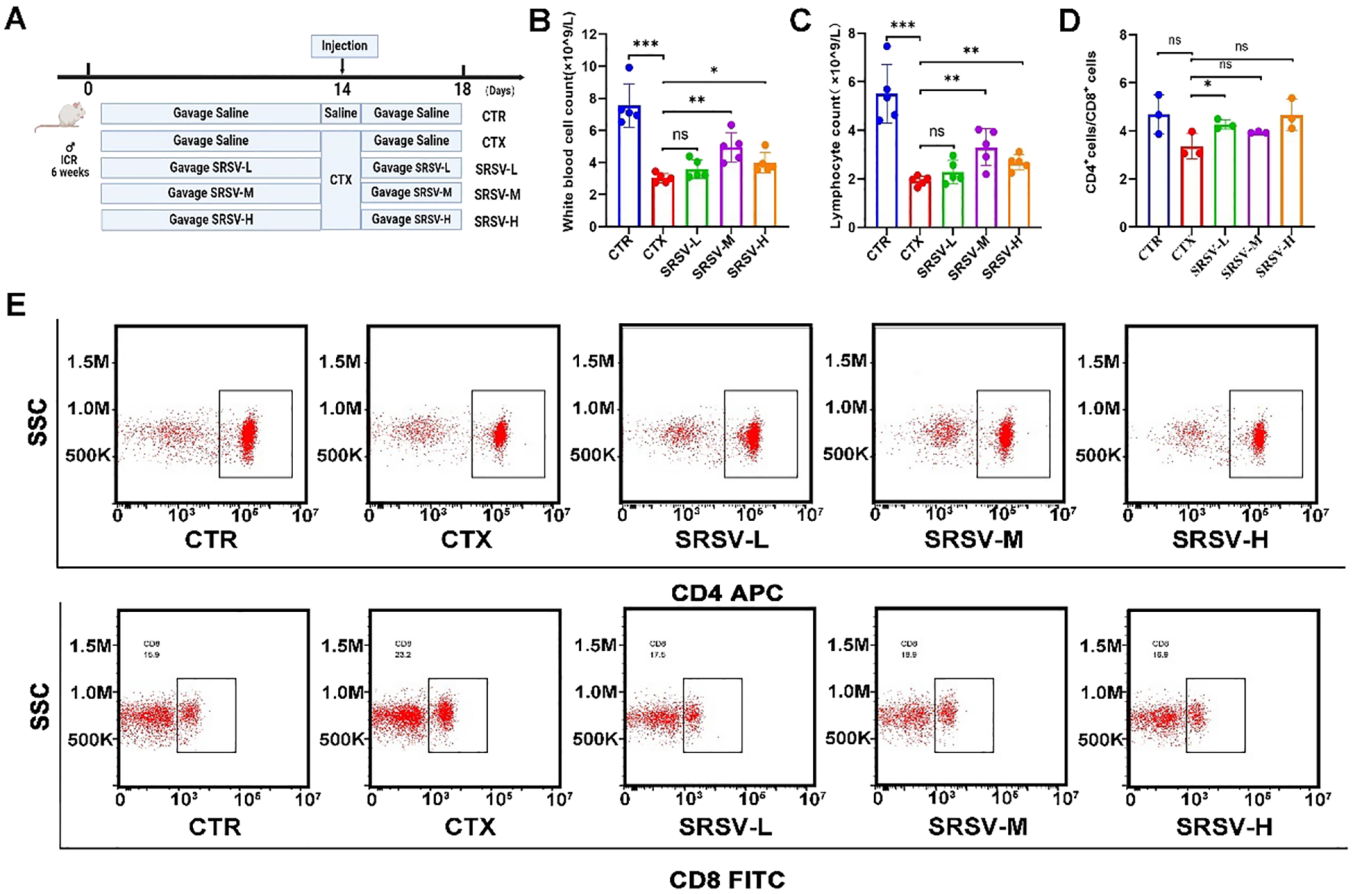
Effects of varying concentrations of SRSV on immune function in immunosuppressed mice. **(A)** Experimental procedure flowchart. Mice in the CTR and CTX groups were gavaged with saline, while the intervention groups received an equivalent volume of SRSV solution prepared in saline. On day 14, all groups except the CTR group were intraperitoneally injected with CTX. Samples were collected on day 18. **(B)** Changes in peripheral blood WBC counts across all groups. **(C)** Changes in peripheral blood lymphocyte count across all groups. **(D, E)** Alterations in the CD4^+^/CD8^+^ T lymphocyte ratio in the spleen following SRSV treatment, with representative flow cytometry images. (n = 5, *P < 0.05, **P < 0.01, ***P < 0.001). ns: not significant (p > 0.05).

Next, we investigated the biological activity of SRSV, specifically its potential to restore immune function in CTX-induced immunosuppressed mice. Mice were gavaged with SRSV solutions at concentrations of 75, 150, and 300 mg/kg from day 1 to day 18. On day 14, all groups except the control group received an intraperitoneal injection of CTX at a dose of 100 mg/kg (**Figure 3A**). During the experiment, mice in the model group exhibited symptoms of appetite loss and lethargy following CTX administration, which were alleviated in the SRSV-treated groups. The primary effect of CTX on the hematologic system was a reduction in the white blood cell (WBC) count. Compared to the CTX group, both the SRSV-M and SRSV-H groups showed significant increases in WBC count (*P* < 0.01 and *P* < 0.05, respectively), with the most pronounced improvement observed in the SRSV-M group (**Figure 3B**). Statistical analysis of WBC classification revealed that both SRSV-M and SRSV-H significantly reversed the CTX-induced reduction in lymphocyte count (*P* < 0.01 and *P* < 0.01), whereas the SRSV-L group showed a modest improvement, which was not statistically significant (**Figure 3C**). T lymphocyte subtyping of spleen samples indicated that CTX treatment led to a reduced CD4^+^/CD8^+^ T lymphocyte ratio, which was reversed by SRSV treatment (**Figures 3D, E**).

**Figure 4:**
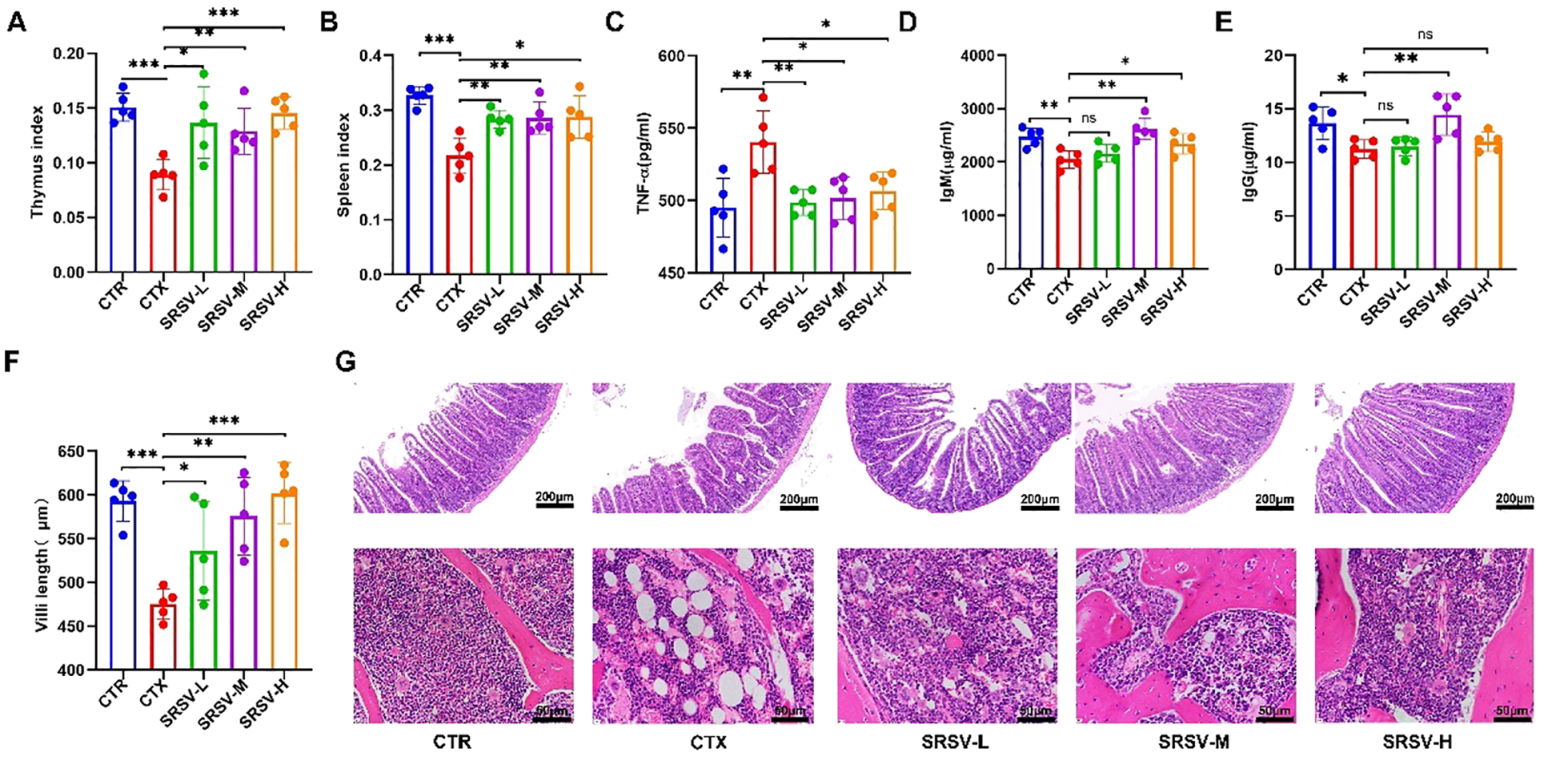
Effects of SRSV intervention on immune organs, bone marrow, and intestinal tissue. **(A)** Changes in the thymus index in each group of mice. **(B)** Changes in the spleen index in each group of mice. **(C)** Serum TNF-α Levels. **(D)** Serum IgM Levels. **(E)** Serum IgG Levels. **(F)** Villus length measurements using CaseViewer 2.4, with statistical analysis. **(G)** Histological analysis. The first row shows jejunum tissue, and the second row shows bone marrow tissue. The white circular areas in the images represent vacuoles observed in yellow bone marrow following HE staining. (n=5, *P < 0.05, **P < 0.01, ***P < 0.001).

The thymus and spleen are crucial immune organs in the body, and the immune organ index is considered a fundamental indicator of immune function (24). Compared to the control group (CTR), both the thymus and spleen indices were significantly reduced in the cyclophosphamide (CTX) group. However, the thymus and spleen indices in the three SRSV-treated groups were significantly higher than those in the CTX group (**Figures 4A, B**), indicating that SRSV effectively mitigates the CTX-induced reduction in immune organ indices and helps prevent immune organ atrophy (**Supplementary Figures S1A, B**). Next, we assessed the impact of SRSV on intestinal damage and hematopoietic function in immunosuppressed mice through histological analysis and serum inflammatory cytokine measurement. Tumor necrosis factor-alpha (TNF-α), a key pro-inflammatory cytokine, is typically maintained at low levels under normal conditions. After CTX treatment, serum TNF-α levels significantly increased. However, SRSV treatment effectively reversed the CTX-induced elevation of TNF-α levels, restoring them to near-normal levels (**Figure 4C**). IgM is typically the first antibody produced during the early stages of infection and serves as an important indicator of the body’s rapid response to pathogens. In contrast, IgG is generated later and provides long-term immune protection. Measuring the levels of IgM and IgG can thus help evaluate both the strength and type of immune response. Figures D and E show the differences in serum IgM and IgG concentrations among the groups. In the SRSV-treated groups, both IgM and IgG levels were elevated compared to the CTX group, with the most pronounced improvement observed in the SRSV-M group. These findings suggest that SRSV treatment can promote the synthesis of IgM and IgG to a certain extent, thereby enhancing long-term immune responses, particularly in the SRSV-M group (**Figures 4D, E**). Histological analysis of the jejunum using HE staining revealed that compared with the normal group, the intestinal villi in the CTX group were damaged by drug-induced side effects, appearing irregular and fragmented, with clearly visible sheet-like lesions at the villus tips. Following intervention with SRSV at three different dosages, the villi appeared relatively intact, and the average villus length was significantly increased compared to the model group (**Figures 4F, G**). As an essential hematopoietic and immune tissue, the bone marrow is often suppressed by prolonged CTX treatment. HE-stained femur sections showed that, following treatment with all three doses of SRSV, bone marrow tissue was denser compared to the CTX group. Additionally, the number of vacuoles in the bone marrow decreased, and the number of nucleated and dividing cells was significantly restored (**Figure 4G**).

### 3.3 SRSV modulates the structure and composition of the GM in mice

Given that oligosaccharides provide a carbon source for specific probiotics and can influence the structure and composition of the GM, we conducted 16S rRNA gene sequencing on fecal samples from CTX-induced immunosuppressed mice to assess how SRSV affects their GM. After investigating the immune-modulatory effects of various SRSV concentrations on immunosuppressed mice, we found that the medium dose (150 mg/kg) yielded the most significant improvement. As a result, we selected the medium dose (150 mg/kg) as the intervention concentration for GM analysis and compared it with the CTR and CTX groups for further examination.

**Figure 5:**
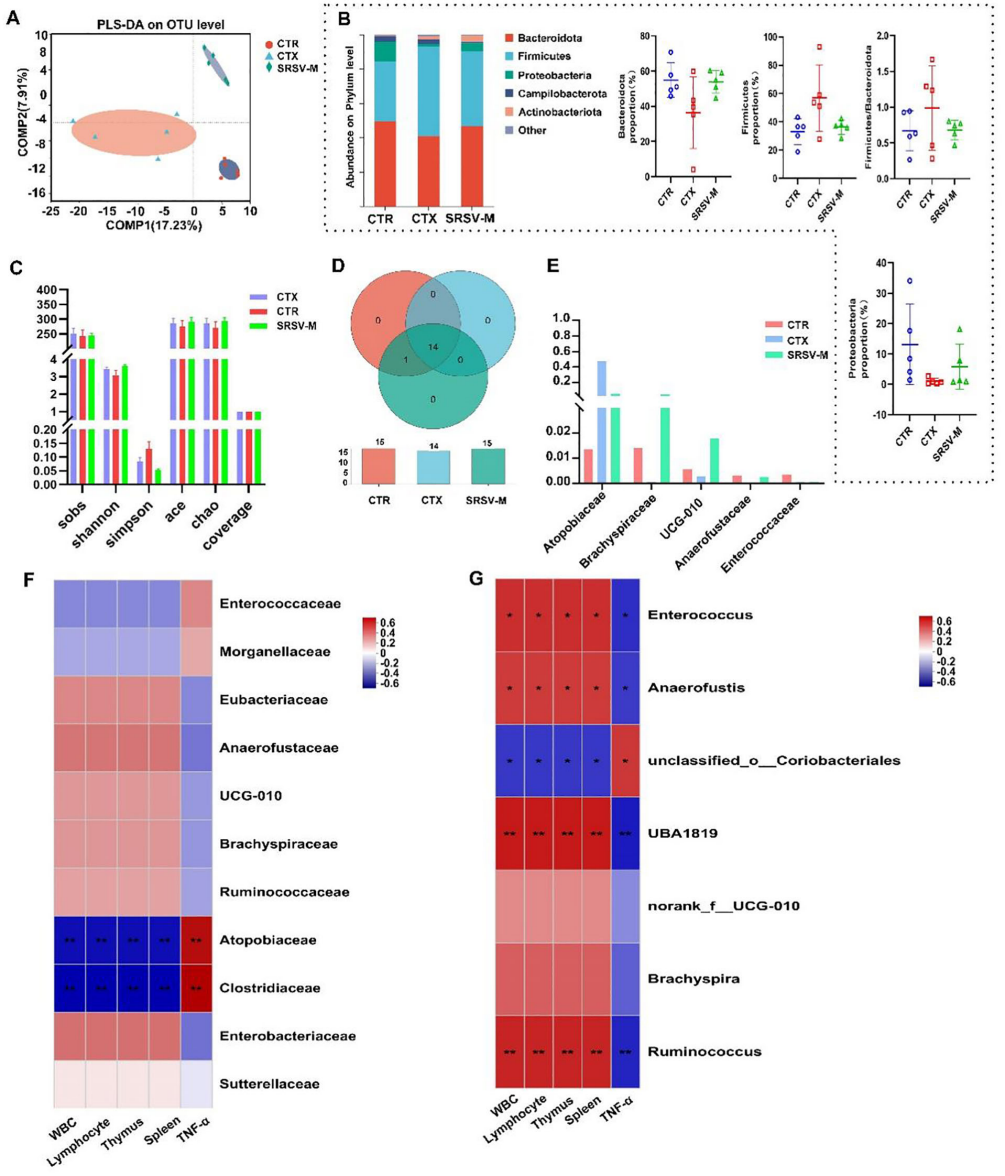
Effects of SRSV on GM. **(A)** Partial least squares discriminant analysis (PLS-DA): a method that reduces within-group variance while maximizing between-group variance, visually displaying group differences along coordinate axes. **(B)** Bar chart of relative abundance at the phylum level: depicts community composition across groups, highlighting changes in dominant microbial phyla. **(C)** Alpha diversity indices: includes Sobs, ACE, Chao, Shannon, Simpson, and coverage indices, reflecting the richness and diversity of GM in fecal samples among groups. **(D)** Venn diagram at the class taxonomic level: illustrates shared and unique species across the experimental groups, providing insights into species overlap and differences. **(E)** Comparison of differentially enriched taxa at the family level: highlights significant variations in the abundance of microbial families across groups. **(F)** Correlation heatmap at the family level: displays associations between differentially abundant microbial families and immune indices, emphasizing positive and negative correlations. **(G)** Correlation heatmap at the genus level: shows relationships between genus-level microbial taxa and immune indicators, identifying key genera associated with improved immune function. n = 5, *P < 0.05, **P < 0.01.

The rarefaction curve (**Supplementary Figure S2A**) demonstrates that the sequencing data captured most of the species diversity, ensuring sufficient coverage for subsequent data analysis. PLS-DA results revealed a distinct separation of the three sample groups along the axes at the operational taxonomic unit (OTU) level, enabling differential analysis between the species groups (**Figure 5A**). **Figure 5B** illustrates the relative abundance of microbial taxa in fecal samples from different groups. At the phylum level, the most abundant microbial phyla across all experimental groups were *Bacteroidota*, *Firmicutes*, and *Proteobacteria*, with *Bacteroidota* being the dominant phylum in the GM of healthy mice. Treatment with CTX disrupted the GM’s original balance, leading to a decrease in the relative abundance of *Bacteroidota* and an increase in the abundance of *Firmicutes*. Changes in high-abundance species are often key drivers of GM alterations, and SRSV was found to reverse the imbalance between the *Bacteroidota* and *Firmicutes* phyla. The α-diversity index is commonly used to describe the richness and diversity of microbial communities. To assess this, we evaluated several common α-diversity indices, including the Sobs, ACE, and Chao indices, to characterize the species richness of the communities. The SRSV-M group showed an increase in all three indices compared to the CTX group, suggesting that SRSV-M supplementation enhanced community richness. The Shannon and Simpson indices are used to assess the diversity of microbial communities. Compared to the CTX group, SRSV treatment increased the Shannon index and decreased the Simpson index (**Figure 5C**), suggesting that SRSV supplementation improved both the richness and diversity of the GM in the CTX-treated mice.

Venn diagrams were used to visualize the shared and unique species across the three groups of samples, providing an intuitive representation of the similarity and overlap in species composition. At the class level, the Venn diagram showed that the CTX group lacked species shared by both the CTR and SRSV groups, such as *c:Cyanobacteriia* (**Figure 5D**). At the family taxonomic level, significant differences were observed in the abundances of *Atopobiaceae*, *Brachyspiraceae*, *UCG-010*, *Anaerofustaceae*, and *Enterococcaceae* across the three groups. Moreover, SRSV treatment was found to alleviate the dysbiosis in the GM induced by CTX, restoring the abundance of these microbial families (**Figure 5E**). A heatmap was used to analyze the correlation between differentially abundant microbial taxa at the family level and immune indicators. The results demonstrated that *Brachyspiraceae* and *Anaerofustaceae* were positively correlated with white blood cell and lymphocyte counts, and negatively correlated with inflammatory cytokine levels (**Figure 5F**). The Kruskal-Wallis rank sum test was used to assess significant intergroup differences. The analysis revealed that *Ruminococcus* (some members of which produce short-chain fatty acids), *UBA1819*, *Anaerofustis*, and other genera were the primary taxa exhibiting significant differences between the CTX group and the other two groups of mice (**Supplementary Figure S2B**). A correlation heatmap was generated to explore the relationship between differentially abundant genera and immune indices. The analysis revealed that genera enriched in the CTR and SRSV-M groups, such as *Ruminococcus*, *Brachyspira*, *UBA1819*, and *Anaerofustis*, showed a positive correlation with improved immune indices (**Figure 5G**). These findings suggest that SRSV intervention alleviates CTX-induced GM dysbiosis by inhibiting the growth of harmful bacteria and promoting the proliferation of beneficial bacteria.

### 3.4 Depletion of the GM in mice significantly compromised the ameliorative effects of SRSV on CTX-induced immunosuppression

**Figure 6:**
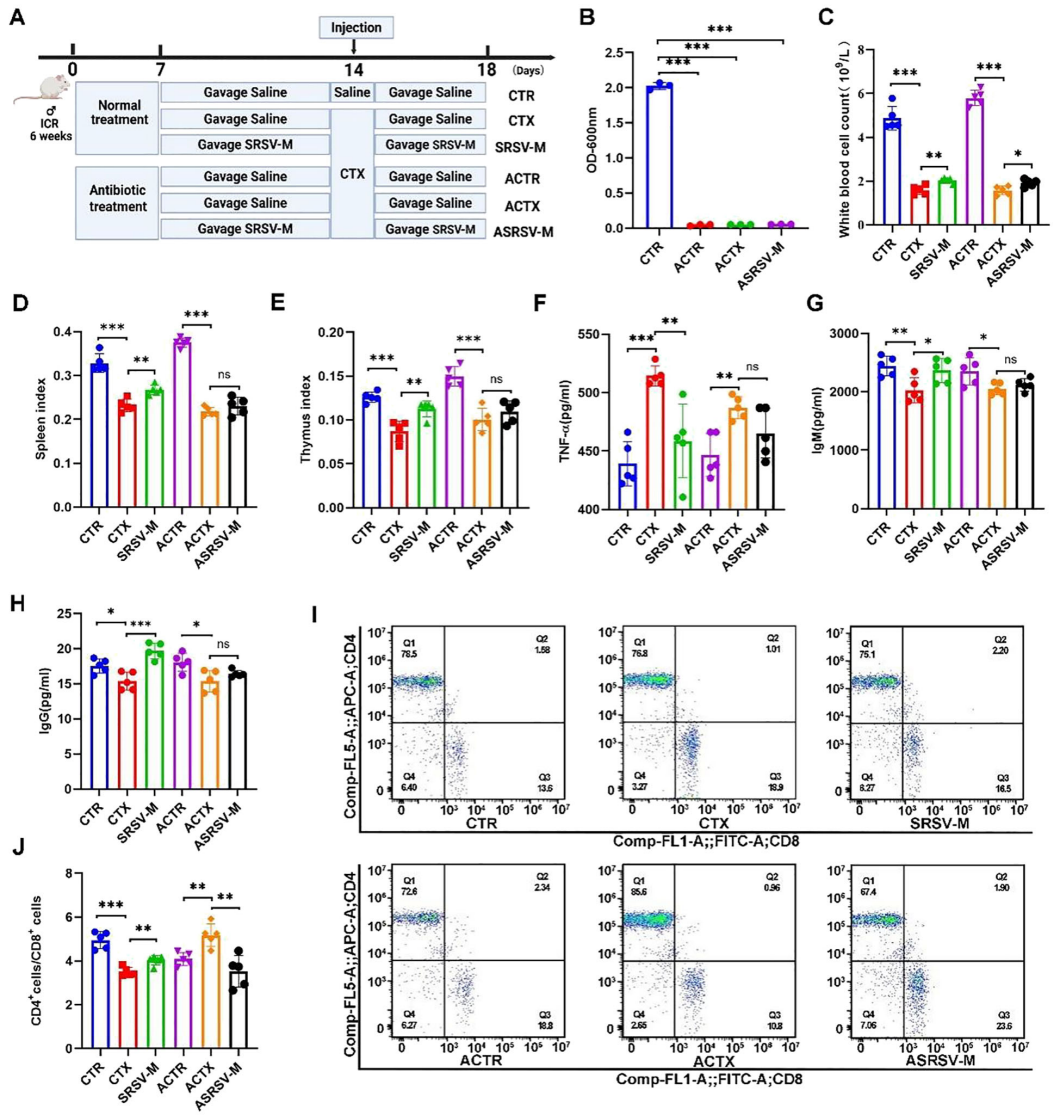
Impact of GM on SRSV efficacy in AIMD mouse models. **(A)** Experimental protocol. Antibiotic-treated mice were categorized into three groups: ACTR (Antibiotic + CTR), ACTX (Antibiotic + CTX), and ASRSV-M (Antibiotic + SRSV-M + CTX). **(B)** OD values of bacterial cultures from fecal samples were measured at 600 nm. **(C)** White blood cell counts. **(D)** Spleen Index **(E)** Thymus index. **(F)** Serum TNF-α Levels. **(G)** Serum IgM Levels. **(H)** Serum IgG Levels. **(I)** Flow cytometry analysis of CD4^+^ and CD8^+^ T cell proportions in the spleen, including representative flow cytometry plots. **(J)** CD4^+^/CD8^+^ T cell ratios. (*P < 0.05, **P < 0.01, ***P < 0.001).

The 16S rRNA sequencing results demonstrated that SRSV alleviated CTX-induced GM dysbiosis in mice. To elucidate the role of GM in SRSV-mediated amelioration of CTX-induced immunosuppression, the AIMD mouse model was established. Antibiotics were administered in sterile water prior to SRSV treatment, and the mice were allowed to drink freely for 7 days (**Figure 6A**). Fecal samples were then collected from both the control group and the antibiotic-treated group for microbial culture. Compared to the control group, the optical density (OD) values of fecal cultures from antibiotic-treated mice were significantly reduced (P < 0.0001) (**Figure 6B**). Solid and liquid culture media were used to cultivate the fecal samples, with the control group exhibiting dense bacterial colonies, whereas the antibiotic-treated group showed almost no colony growth (**Supplementary Figures S3A, B**). These findings confirm that the antibiotic treatment protocol successfully depleted the GM, thereby establishing the AIMD mouse model for subsequent experiments.

After CTX administration, the peripheral blood white blood cell count in mice significantly decreased; however, this effect was notably alleviated by SRSV intervention. In contrast, in antibiotic-treated mice that were subsequently given SRSV (Antibiotic+SRSV-M+CTX, ASRSV-M), the increase in white blood cell count was less pronounced compared to the SRSV-M group (**Figure 6C**). The immune organ indices of the spleen and thymus demonstrated that, under the influence of the GM, SRSV intervention mitigated the decline in immune organ indices induced by CTX. However, the increase in immune organ indices in the ASRSV-M group of mice was not significantly different (**Figures 6D, E**). SRSV-M treatment significantly reduced TNF-α levels and alleviated CTX-induced inflammation, indicating that SRSV-M has anti-inflammatory properties. However, in the antibiotic-treated ASRSV-M group, TNF-α levels were not significantly decreased, suggesting that the GM plays a critical role in the anti-inflammatory effects of SRSV (**Figure 6F**). Furthermore, the levels of IgM and IgG in the SRSV-M group were significantly higher than those in the CTX group, indicating that SRSV effectively enhances the immune response. In contrast, no significant increase in IgM and IgG levels was observed in the ASRSV-M group, where the GM had been depleted, further supporting the notion that the immune-enhancing effects of SRSV are weakened following depletion of the GM (**Figures 6G, H**). In healthy mice, the SRSV-M group improved immune function by restoring CD4^+^ T cell activity, resulting in a recovery of the CD4^+^ T/CD8 ^+^ T ratio. In contrast, in the ACTX group, antibiotic intervention disrupted immune homeostasis by eliminating the gut microbiota, resulting in abnormal proliferation of CD4^+^ T cells and an elevated CD4^+^/CD8^+^ T cell ratio. This imbalance led to immune dysregulation and aberrant immune responses. Nevertheless, in the ASRSV-M group, this dysregulation was alleviated, suggesting that SRSV-M treatment may help restore GM balance to a certain extent (**Figures 6I, J**). These findings indicate that the immunoenhancing effects of SRSV in immunosuppressed mice are diminished when the GM is depleted, compared to mice with an intact microbial community. This attenuation may result from the disruption of the GM’s essential role in immune modulation due to antibiotic intervention. Nonetheless, SRSV was still capable of partially mitigating the adverse effects associated with microbial dysbiosis, suggesting that its immunomodulatory activity involves not only direct regulation of the immune system, but also the restoration of immune function and homeostasis through modulation of the GM.

### 3.5 SRSV promotes the production of immune-related gut microbial metabolites

The above results demonstrate that GM plays an important role in the immunomodulatory effects of SRSV. However, the interaction between the GM and the host is predominantly mediated by microbial metabolites. These metabolites can modulate endogenous signaling pathways, serve as nutritional substrates for host cells, and influence the intestinal microenvironment, thereby playing a pivotal role in host-microbiota communication and immune regulation (25). Certain gut microbial metabolites, including short-chain fatty acids (SCFAs), tryptophan, and secondary bile acids, have been shown to regulate the host immune system and intestinal barrier function (26). Based on this, we hypothesize that in this study, metabolites produced by the GM under the influence of SRSV may act as chemical messengers, facilitating the interaction between the microbiota and the host. To investigate this, we conducted untargeted metabolomics analysis via liquid chromatography-mass spectrometry (LC-MS) on fecal samples from the CTR, CTX, and SRSV-M groups of mice.

**Figure 7:**
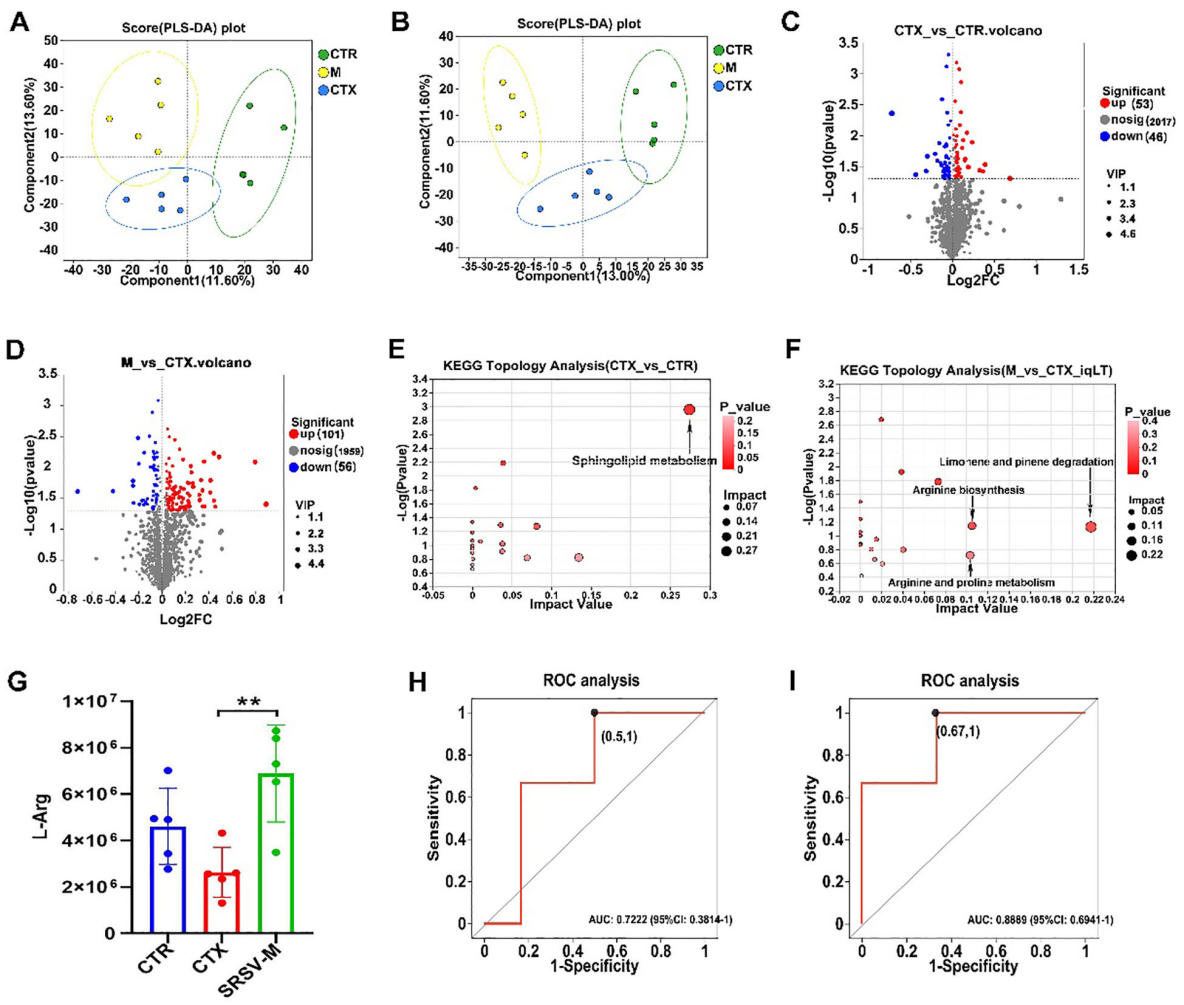
Untargeted metabolomics analysis. **(A)** PLS-DA score plot under positive ion mode. **(B)** PLS-DA score plot under negative ion mode. **(C)** Volcano plot of differential metabolites between the CTX and CTR groups. **(D)** Volcano plot of differential metabolites between the SRSV-M and CTX groups. **(E)** KEGG pathway differential analysis between the CTX and CTR groups. **(F)** KEGG pathway differential analysis between the SRSV-M and CTX groups. In the pathway enrichment analysis, each bubble represents a KEGG pathway, and the size of the bubble indicates the impact value; larger bubbles represent more important pathways. **(G)** Analysis of L-Arg expression levels among the three groups (n=5, **P < 0.01). **(H)** ROC curve for L-Arg in the CTX vs. CTR comparison (AUC > 0.7). **(I)** ROC curve for L-Arg in the SRSV-M vs. CTX comparison (AUC > 0.7).

Orthogonal Partial Least Squares Discriminant Analysis (PLS-DA) score plots demonstrated clear segregation among the three experimental groups (**Figures 7A, B**), with model validation confirming robustness and the absence of overfitting (**Supplementary Figures S4A, B**). These findings validate the experimental design and grouping, supporting subsequent analyses. Metabolomics profiling identified metabolites across eight major categories, including vitamins, cofactors, and peptides (**Supplementary Figure S4C**). Differential metabolites were identified based on Variable Importance in Projection (VIP) values from PLS-DA, fold change, and p-values from univariate analysis, and the results were visualized using volcano plots. The volcano plots revealed that, compared to the CTR group, 53 metabolites were upregulated and 46 downregulated in the CTX group. Relative to the CTX group, 101 metabolites were upregulated and 56 downregulated in the SRSV-M group (**Figures 7C, D**). To further investigate the metabolic pathways regulated by SRSV, pathway analysis revealed that sphingolipid metabolism was significantly altered between the CTR and CTX groups. Specifically, the CTX group exhibited increased levels of the metabolites N-palmitoyl sphingosine and phytosphingosine within this pathway (**Figure 7E**). Additionally, significant differences in the arginine biosynthesis and arginine-proline metabolism pathways were observed between the CTX and SRSV-M groups (**Figure 7F**). L-arginine (L-Arg) was identified as the key differential metabolite in these pathways. A separate analysis of L-Arg expression across all three groups showed that L-Arg levels in the CTX group were significantly lower than those in the CTR and SRSV-M groups (**Figure 7G**). To further explore the relevance of L-Arg, Receiver Operating Characteristic (ROC) curve analysis was performed. The ROC analysis revealed that the Area Under the Curve (AUC) values for both CTX vs. CTR and SRSV-M vs. CTX comparisons exceeded 0.7, indicating strong differentiation between the groups (**Figures 7H, I**). These findings suggest that L-Arg, as a GM-derived metabolite, is enriched following SRSV treatment, and its associated metabolic pathway is significantly enhanced. This implies that enhancing L-Arg production by the GM may be a potential mechanism through which SRSV exerts its immune-enhancing effects.

### 3.6 L-Arg improves immune function in immunosuppressed mice

**Figure 8:**
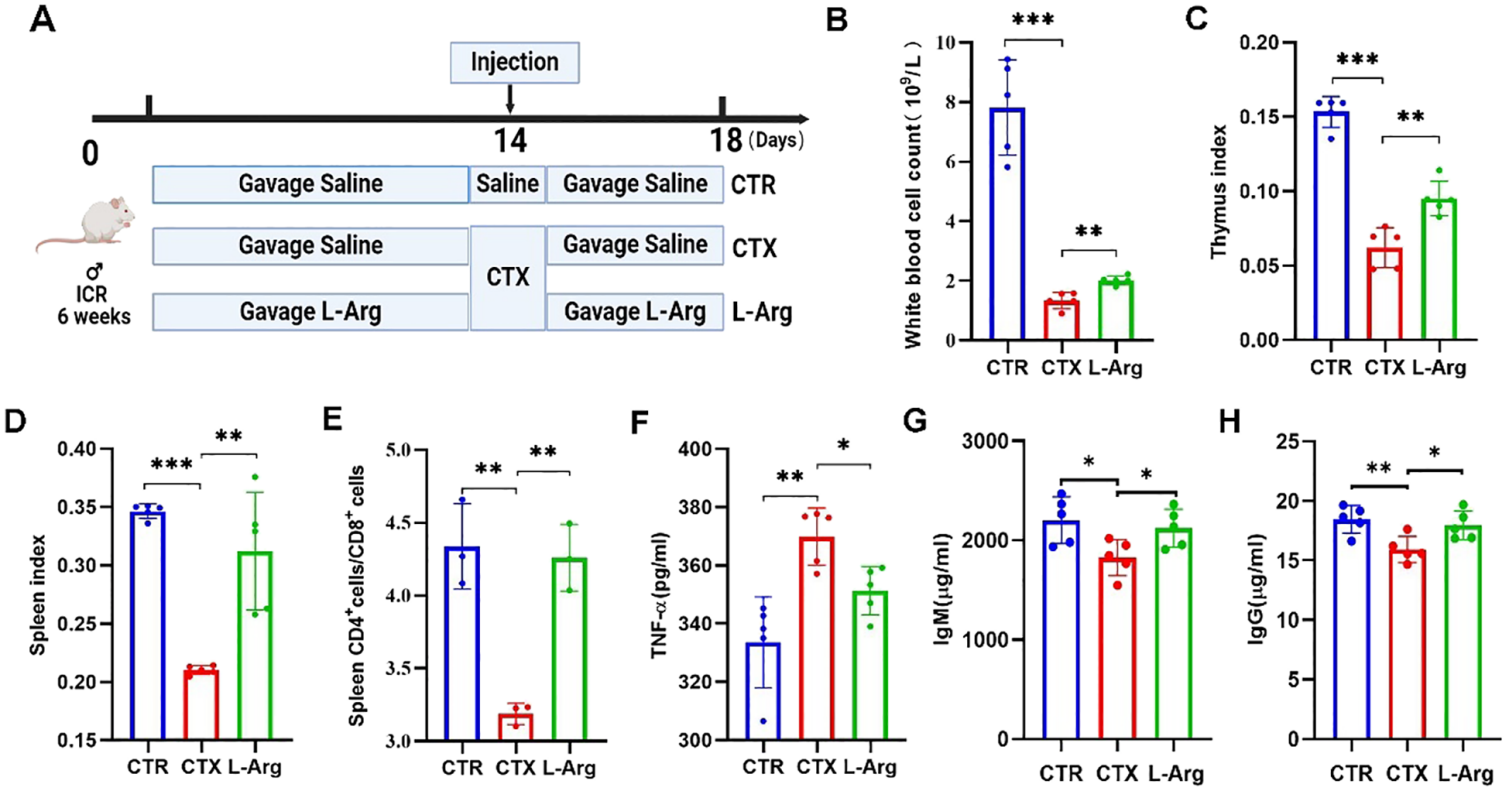
Impact of L-Arg on immune function in immunosuppressed mice. **(A)** Experimental protocol. The CTR and CTX groups were orally gavaged with saline, while the L-Arg intervention group received L-Arg in saline. CTX was administered intraperitoneally on day 14, with samples collected on day 18. **(B)** White blood cell counts. **(C)** Thymus Index. **(D)** Spleen Index. **(E)** Spleen CD4+/CD8+ T Cell Ratio. **(F)** Serum TNF-α Levels. **(G)** Serum IgM Levels. **(H)** Serum IgG Levels. (n=5; *P < 0.05, **P < 0.01, ***P < 0.001).

To verify the aforementioned hypothesis, immunosuppressed mice were fed L-Arg alone to investigate whether it could enhance immune function. L-Arg was administered at a concentration of 500 mg/kg, following the protocol established for the SRSV intervention experiment (**Figure 8A**). The results showed that L-Arg alleviated the CTX-induced reduction in peripheral blood leukocyte counts in mice (**Figure 8B**), achieving efficacy comparable to that of SRSV (**Figure 3B**). Similarly, the decreases in thymus and spleen indices caused by CTX were improved with L-Arg treatment (**Figures 8C, D**). The CTX-induced decline in the spleen CD4+ T cell/CD8+ T cell ratio was reversed by L-Arg administration (**Figure 8E**). Serum analysis further demonstrated that L-Arg treatment significantly reduced CTX-induced elevations in TNF-α levels (**Figure 8F**), indicating its potential anti-inflammatory effects in suppressing CTX-triggered inflammatory responses. Additionally, L-Arg mitigated the CTX-induced reductions in IgM and IgG levels (**Figures 8G, H**). These findings confirm that L-Arg can ameliorate CTX-induced immunosuppression with effects comparable to those of SRSV, supporting the hypothesis that SRSV enhances immune function in chemotherapy-induced immunosuppression by modulating the GM to increase L-Arg production.

## 4 Discussion

Prebiotics are organic compounds that are not digested or absorbed by the host. They selectively promote the growth and proliferation of beneficial gut microorganisms and modulate the immune system, thereby enhancing the overall health of the host (27, 28). Common prebiotics are primarily oligosaccharides, largely derived from plants, especially edible herbs (29). In this study, a mixture of oligosaccharides (SRSV) was obtained from the aqueous extract of *Dolichos lablab L.* through isolation and purification. The main components of SRSV include sucrose, raffinose, stachyose, and verbascose. Among these, raffinose, stachyose, and verbascose are recognized as prebiotics, and their probiotic effects have been widely documented. For example, stachyose has been shown to regulate GM balance and, as a prebiotic, increase the relative abundance of beneficial bacteria such as *Bifidobacterium* and *Lactobacillus (* 30). Additionally, it enhances the abundance of *Akkermansia*, which exhibits anti-inflammatory effects, particularly in DSS-induced colitis models in mice (31, 32). Raffinose has shown promising potential in improving GM balance and enhancing gut immunity. Its effects include increasing the abundance of beneficial bacteria (33), strengthening the intestinal barrier (34), promoting epithelial cell renewal (35), and modulating immune responses and inflammation (36). Verbascose, on the other hand, has been demonstrated to enhance the phagocytic activity of peritoneal macrophages, stimulate the release of nitric oxide and a variety of cytokines, and exhibit potential immune-boosting effects. These properties of raffinose and verbascose suggest their valuable roles as prebiotics, contributing to both gut health and overall immune function (37). Additionally, Yang et al. reported a novel oligosaccharide preparation (DSG) consisting of 55.3% stachyose, 25.8% raffinose, and 9.7% verbascose. This formulation was shown to promote the growth of beneficial gut bacteria, suppress pathogenic bacteria, enhance intestinal motility, and facilitate fecal excretion, thereby improving gut health and alleviating constipation (38). The above studies suggest that raffinose, stachyose, and verbascose exhibit potential benefits in enhancing gut health, mitigating inflammatory responses, regulating immune function, and modulating GM composition. However, their potential to alleviate chemotherapy-induced immunosuppression remains unexplored.

The present experimental study confirmed that early intervention with SRSV successfully prevented the CTX-induced reduction in peripheral blood leukocyte counts in mice. Peripheral blood leukocytes play a pivotal role in the body’s immune defense (39). The bone marrow serves as a critical site for both hematopoiesis and immune regulation (40). CTX induces bone marrow suppression, impairing hematopoietic function. This is primarily manifested as a reduction in white blood cells and neutrophils. Subsequently, the proliferation and differentiation of hematopoietic stem cells (HSCs) are inhibited, leading to a decrease in red blood cells and platelets (41). In our study, CTX administration also led to a reduction in red blood cells and platelets. To investigate whether SRSV could alleviate CTX-induced bone marrow damage, femoral HE staining was conducted on mice. Bone marrow sections from the SRSV-treated group exhibited a significantly higher red-to-yellow marrow ratio compared to the CTX model group, suggesting that SRSV protects hematopoietic and immune functions from CTX-induced suppression. As critical immune organs, the thymus and spleen indices are direct indicators of nonspecific immunity and reflect the organism’s overall immune functionality (42). The indices of immune organs are positively correlated with the quantity of immune cells residing within these organs. T lymphocytes, which play a central role in cellular immunity, are marked by the broad expression of CD3^+^ T cells, on mature T cells, serving as a key indicator of immune status. Within this population, CD4^+^ T cells facilitate supportive roles during immune responses, whereas CD8^+^ T cells function as the primary effectors of cellular immunity, efficiently eliminating invading pathogens. The CD4^+^/CD8^+^ T lymphocytes remain in equilibrium in a healthy state, and any changes in this ratio may indicate a disturbance in cellular immunity (43). This is consistent with the results of the present study, where early intervention with SRSV protected the spleen and thymus. Immune organ indices did not show significant reductions, and the decrease in the CD4^+^/CD8^+^ T cell ratio in the spleen was significantly ameliorated.

The GM functions as a signaling hub, integrating dietary, environmental, genetic, and immune signals to modulate host metabolism and immunity (44). The intestinal mucosal immune system, which includes lymph nodes, the lamina propria, and epithelial cells, serves as a protective barrier to maintaining intestinal integrity. Our research demonstrated that the intestinal damage caused by CTX injection was alleviated to some extent by SRSV pre-intervention, as evidenced by the preservation of intestinal villi and the reduction of inflammatory factors in the blood. The GM is maintained under the constant monitoring of the mucosal immune system. When alterations in microbiota composition or metabolic activity are detected, the innate immune system communicates adaptive signals to the host, enabling adjustments in the microbiota’s composition and function (45). In the present study, SRSV effectively mitigated the CTX-induced shift in the Firmicutes-to-Bacteroidetes (F/B) ratio. Firmicutes are primarily involved in protein metabolism, whereas Bacteroidetes specialize in metabolizing fibers and polysaccharides. Unlike proteins, glycans are not directly encoded in DNA but are synthesized through enzymatic pathways encoded by specific genes. Bacteroidetes leverage a diverse array of enzymes to degrade and metabolize polysaccharides (46, 47). Alterations in the Bacteroidetes-to-Firmicutes (F/B) ratio can disrupt intestinal absorption and nutrient utilization, ultimately impacting overall health. Research on ethanol-precipitated polysaccharides from Dendrobium officinale (EPDO) demonstrated that EPDO enhanced fatigue resistance in mice by increasing the F/B ratio in the GM. These findings suggest that regulating the F/B ratio may contribute to improved metabolic efficiency and overall host health (48). The interplay between GM and the host is crucial for maintaining overall health. Disruptions in the GM can weaken the intestinal barrier and disturb mucosal immune balance, resulting in increased translocation of microbial components and metabolites. This may lead to systemic immune alterations and metabolic dysregulation. GM dysbiosis, often marked by a reduction in beneficial symbiotic bacteria and an overgrowth of opportunistic pathogens, is commonly associated with the onset and progression of various diseases. The 16S rRNA sequencing results in our study demonstrated that *c:Cyanobacteriia*, a bacterial class present in the intestines of normal mice, disappeared following CTX treatment, indicating significant dysbiosis. Remarkably, pre-administration of SRSV preserved this bacterial class, enabling the continued production of its associated metabolites. Previous studies on Pterocarpus polysaccharides have linked *Cyanobacteriia* with the SCFAs, highlighting their potential role in maintaining gut health and metabolic stability (49). SCFAs are produced by gut bacteria through the degradation and fermentation of undigested carbohydrates by active enzymes (50). In this experiment, the intervention with SRSV prevented the significant reduction of *Spirochetes*, which are SCFA producers, caused by CTX treatment. LEfSE analysis showed significant enrichment of the pathogenic genus *Atopobiaceae* in the CTX group. In contrast, SRSV treatment increased the abundance of beneficial genera such as *Ruminococcus* and *Anaerofustis*. Notably, members of the genus *Ruminococcus* include SCFA producers known for their anti-inflammatory properties (51). Validation experiments using AIMD mice further revealed that the immune protective effects of SRSV were significantly diminished when GM were depleted using antibiotics. This finding is consistent with earlier research highlighting the role of GM in enhancing immune function and resistance (52, 53).

To validate the hypothesis that SRSV exerts immunoprotective effects by modulating the intestinal flora and influencing metabolite production, further non-targeted metabolomics analyses were conducted using mouse fecal samples. Metabolites exhibiting significant intergroup differences and consistently high expression were identified as potential contributors to key metabolic pathways, likely playing critical biological roles in the observed effects. In this study, KEGG pathway enrichment analysis of these differential metabolites revealed that the arginine biosynthesis pathway was significantly enriched in the SRSV-treated group. Moreover, SRSV administration effectively reversed the CTX-induced reduction in L-Arg levels. In anti-tumor responses, L-Arg plays a crucial role in promoting T-cell metabolism and enhancing survival fitness (54). Previous studies have shown that in the MC38 mouse tumor model, both oral administration of L-Arg and intratumoral injection of engineered bacteria producing L-Arg can enhance the tumor-suppressive effects of PD-L1 antibodies (55). In our experiment, feeding immunosuppressed mice with L-Arg alone produced the same immune-enhancing effects as SRSV. Furthermore, correlation analysis between differential metabolites and immune indicators revealed a positive correlation between L-Arg levels and improved immune function in the mice. In conclusion, SRSV’s regulation of GM enhances the production of anti-inflammatory and immune-enhancing metabolites, particularly through the enrichment of the L-Arg metabolism pathway, which may represent a key mechanism through which SRSV exerts its immune-regulating effects.

## 5 Conclusion

**Figure 9:**
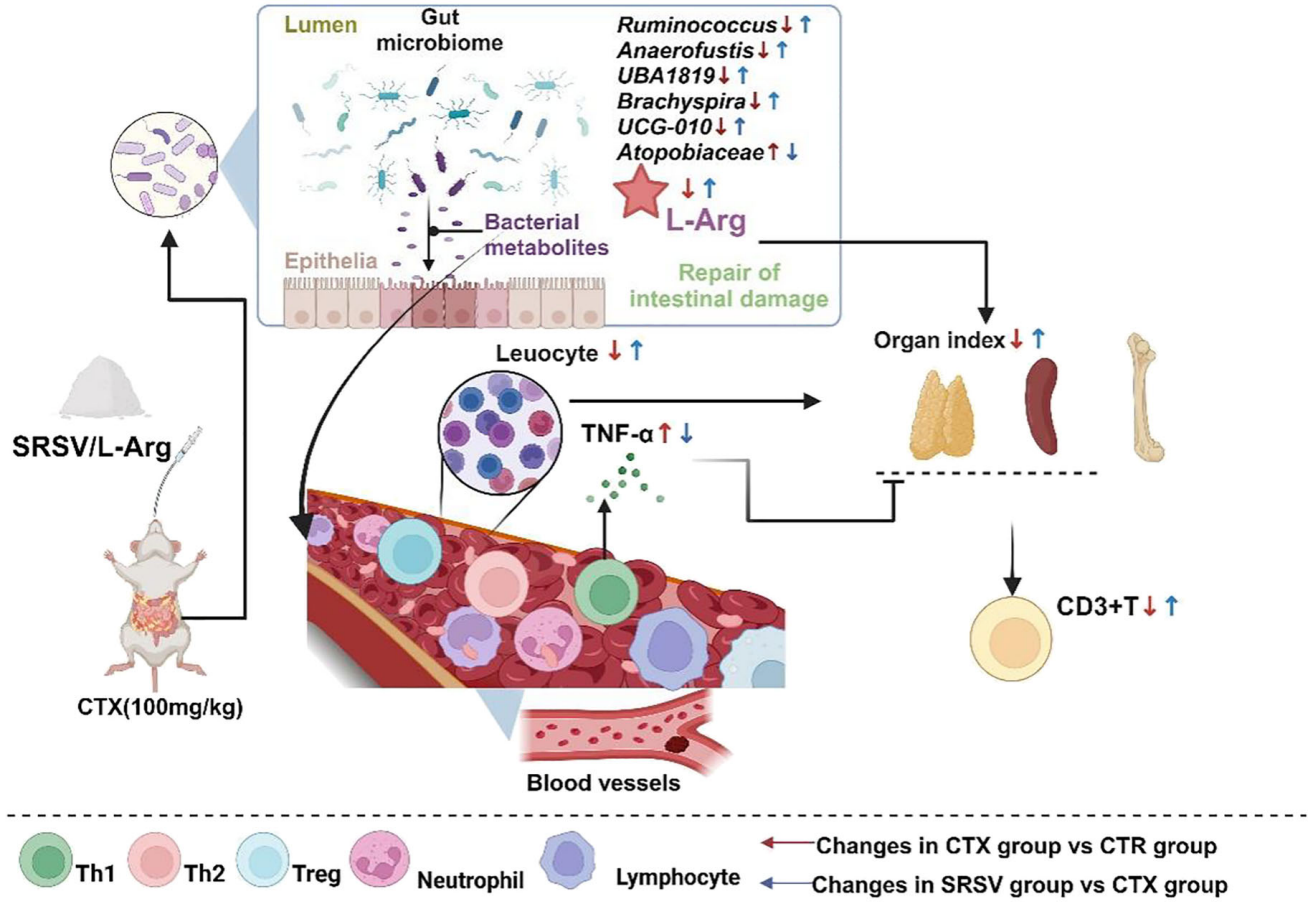
The mechanism by which SRSV protects mice from the side effects of CTX treatment. SRSV acts on the gut microbiome to resist the damage caused by CTX to the gut microenvironment, keeping the gut microbiome healthy and protecting the integrity of the intestinal barrier. Healthy GM produce beneficial metabolite L-Arg, which is transported to various parts of the body, reducing inflammation and enhancing the immune organs’ immune resistance to CTX, thereby avoiding the reduction in peripheral blood white blood cell counts and shrinkage of immune organs.

This study successfully purified and identified oligosaccharides extracted from *Dolichos lablab L.*, determining that the main components of the mixture are sucrose, raffinose, stachyose, and verbascose. The purified oligosaccharide product, SRSV, demonstrated significant efficacy in alleviating immunosuppression induced by the chemotherapeutic drug CTX. Mechanistically, SRSV regulates GM structure and composition, increasing the abundance of beneficial bacteria while suppressing pathogenic bacteria. Furthermore, SRSV modulates the arginine biosynthesis and metabolic pathways, enhancing the production of L-Arg, a metabolite crucial for immune protection (**Figure 9**). These findings present a promising strategy for mitigating chemotherapy-associated side effects in clinical applications and offer new perspectives for the development and use of medicinal food-grade Chinese herbal remedies.

## Data Availability

The datasets presented in this study can be found in online repositories. The names of the repository/repositories and accession number(s) can be found in the article/**Supplementary Material**.

## References

[1] Bray F Ferlay J Soerjomataram I Siegel RL Torre LA Jemal A. Global cancer statistics 2018: GLOBOCAN estimates of incidence and mortality worldwide for 36 cancers in 185 countries. CA: A Cancer J Clin. (2018) 68:394–424. doi: 10.3322/caac.21492, PMID: 30207593

[2] Sung H Ferlay J Siegel RL Laversanne M Soerjomataram I Jemal A. Global cancer statistics 2020: GLOBOCAN estimates of incidence and mortality worldwide for 36 cancers in 185 countries. CA: A Cancer J Clin. (2021) 71:209–49. doi: 10.3322/caac.21660, PMID: 33538338

[3] Pérez-Herrero E Fernández-Medarde A. Advanced targeted therapies in cancer: Drug nanocarriers, the future of chemotherapy. Eur J Pharmaceutics Biopharmaceutics. (2015) 93:52–79. doi: 10.1016/j.ejpb.2015.03.018, PMID: 25813885

[4] Chen S Zhu Y Xu Q Jiang Q Chen D Chen T. Photocatalytic glucose depletion and hydrogen generation for diabetic wound healing. Nat Commun. (2022) 13:5684. doi: 10.1038/s41467-022-33475-7, PMID: 36167814 PMC9515190

[5] Zhu G Luo J Du H Jiang Y Tu Y Yao Y. Ovotransferrin enhances intestinal immune response in cyclophosphamide-induced immunosuppressed mice. Int J Biol Macromolecules. (2018) 120:1–9. doi: 10.1016/j.ijbiomac.2018.08.058, PMID: 30114420

[6] Yang LS Cameron K Papaluca T Basnayake C Jackett L McKelvie P. Cyclophosphamide-associated enteritis: A rare association with severe enteritis. World J Gastroenterol. (2016) 22:8844–8. doi: 10.3748/wjg.v22.i39.8844, PMID: 27818600 PMC5075559

[7] Yoshida S Temmoku J Asano T Iwasaki T Matsumoto H Fujita Y. Severe enteritis after cyclophosphamide administration in a patient with microscopic polyangiitis: A case report and literature review. Internal Med. (2023) 62:1279–85. doi: 10.2169/internalmedicine.0434-22, PMID: 36104200 PMC10208791

[8] Vogt L Meyer D Pullens G Faas M Smelt M Venema K. Immunological properties of inulin-type fructan. Crit Rev Food Sci Nutr. (2015) 55:414–36. doi: 10.1080/10408398.2012.656772, PMID: 24915372

[9] Zhang N Jin M Wang K Zhang Z Shah NP Wei H. Functional oligosaccharide fermentation in the gut: Improving intestinal health and its determinant factors-A review. Carbohydr Polymers. (2022) 284:119043. doi: 10.1016/j.carbpol.2021.119043, PMID: 35287885

[10] Reens AL Cosetta CM Saur R Trofimuk O Brooker SL Lee ML. Tunable control of B. infantis abundance and gut metabolites by co-administration of human milk oligosaccharides. Gut Microbes. (2024) 16:2304160. doi: 10.1080/19490976.2024.2304160, PMID: 38235736 PMC10798361

[11] Autran CA Kellman BP Kim JH Asztalos E Blood AB Spence ECH. Human milk oligosaccharide composition predicts risk of necrotising enterocolitis in preterm infants. Gut. (2018) 67:1064–70. doi: 10.1136/gutjnl-2016-312819, PMID: 28381523

[12] Morozov V Hansman G Hanisch FG Schroten H Kunz C. Human milk oligosaccharides as promising antivirals. Mol Nutr Food Res. (2018) 62:e1700679. doi: 10.1002/mnfr.201700679, PMID: 29336526

[13] Hu B Ye C Leung EL-H Zhu L Hu H Zhang Z. Bletilla striata oligosaccharides improve metabolic syndrome through modulation of gut microbiota and intestinal metabolites in high fat diet-fed mice. Pharmacol Res. (2020) 159:104942. doi: 10.1016/j.phrs.2020.104942, PMID: 32504835

[14] Zhang Z Lu W Liu P Li M Ge X Yu B. Microbial modifications with Lycium barbarum L. oligosaccharides decrease hepatic fibrosis and mitochondrial abnormalities in mice. Phytomedicine. (2023) 120:155068. doi: 10.1016/j.phymed.2023.155068, PMID: 37690228

[15] Rana M Jassal S Yadav R Sharma A Puri N Mazumder K. Functional β-mannooligosaccharides: Sources, enzymatic production and application as prebiotics. Crit Rev Food Sci Nutr. (2023) 64:10221–38. doi: 10.1080/10408398.2023.2222165, PMID: 37335120

[16] Zhang T Cheng T Geng S Mao K Li X Gao J. Synbiotic combination between lactobacillus paracasei VL8 and mannan-oligosaccharide repairs the intestinal barrier in the dextran sulfate sodium-induced colitis model by regulating the intestinal stem cell niche. J Agric Food Chem. (2024) 72:2214–28. doi: 10.1021/acs.jafc.3c08473, PMID: 38237048

[17] Chen S-M Zeng F-S Fu W-W You H-T Mu X-Y Chen G-F. White hyacinth bean polysaccharide ameliorates diabetes via microbiota-gut-brain axis in type 2 diabetes mellitus rats. Int J Biol Macromolecules. (2023) 253:27307. doi: 10.1016/j.ijbiomac.2023.127307, PMID: 37813213

[18] Gao Y Huang R Qiu Y Liu Y Chen L. Characterization of the chemical composition of different parts of Dolichos lablab L. and revelation of its anti-ulcerative colitis effects by modulating the gut microbiota and host metabolism. J Ethnopharmacology. (2024) 322:117629. doi: 10.1016/j.jep.2023.117629, PMID: 38135234

[19] Paviani B Masarweh C Bhattacharya M Ozturk G Castillo J Couture G. Eat your beets: Conversion of polysaccharides into oligosaccharides for enhanced bioactivity. Int J Biol Macromolecules. (2024) 256:128472. doi: 10.1016/j.ijbiomac.2023.128472, PMID: 38029906

[20] DuBois M Gilles KA Hamilton JK Rebers PA Smith F. Colorimetric method for determination of sugars and related substances. Analytical Chem. (2002) 28:350–60. doi: 10.1021/ac60111a017

[21] Jiang Y Shang Z Lv X Du M Ma L Hou G. Structure elucidation and antitumor activity of a water soluble polysaccharide from Hemicentrotus pulcherrimus. Carbohydr Polymers. (2022) 292:119718. doi: 10.1016/j.carbpol.2022.119718, PMID: 35725190

[22] Elango D Rajendran K van der Laan L Sebastiar S Raigne J Thaiparambil NA. Raffinose family oligosaccharides: friend or foe for human and plant health? Front Plant Sci. (2022) 13:829118. doi: 10.3389/fpls.2022.829118, PMID: 35251100 PMC8891438

[23] MyIntyre DD Vogel HJ. Complete assignment of the 1H-NMR spectrum of stachyose by two-dimensional NMR spectroscopy. J Natural Products. (1989) 52:1008–14. doi: 10.1021/np50065a015

[24] Sun S Li B Wu M Deng Y Li J Xiong Y. Effect of dietary supplemental vitamin C and betaine on the growth performance, humoral immunity, immune organ index, and antioxidant status of broilers under heat stress. Trop Anim Health Production. (2023) 55:96. doi: 10.1007/s11250-023-03500-y, PMID: 36823253

[25] Sonnenburg JL Bäckhed F. Diet–microbiota interactions as moderators of human metabolism. Nature. (2016) 535:56–64. doi: 10.1038/nature18846, PMID: 27383980 PMC5991619

[26] Nicolas GR Chang PV. Deciphering the chemical lexicon of host–gut microbiota interactions. Trends Pharmacol Sci. (2019) 40:430–45. doi: 10.1016/j.tips.2019.04.006, PMID: 31079848 PMC6681900

[27] Gibson GR Hutkins R Sanders ME Prescott SL Reimer RA Salminen SJ. Expert consensus document: The International Scientific Association for Probiotics and Prebiotics (ISAPP) consensus statement on the definition and scope of prebiotics. Nat Rev Gastroenterol Hepatol. (2017) 14:491–502. doi: 10.1038/nrgastro.2017.75, PMID: 28611480

[28] Yadav MK Kumari I Singh B Sharma KK Tiwari SK. Probiotics, prebiotics and synbiotics: Safe options for next-generation therapeutics. Appl Microbiol Biotechnol. (2022) 106:505–21. doi: 10.1007/s00253-021-11646-8, PMID: 35015145 PMC8749913

[29] Zhang C Pi X Li X Huo J Wang W. Edible herbal source-derived polysaccharides as potential prebiotics: Composition, structure, gut microbiota regulation, and its related health effects. Food Chem. (2024) 458:140267. doi: 10.1016/j.foodchem.2024.140267, PMID: 38968717

[30] Li T Lu X Yang X. Evaluation of clinical safety and beneficial effects of stachyose-enriched α-galacto-oligosaccharides on gut microbiota and bowel function in humans. Food Funct. (2017) 8:262–9. doi: 10.1039/c6fo01290f, PMID: 28001151

[31] He Q He L Zhang F Jian Z Sun J Chen J. Stachyose modulates gut microbiota and alleviates dextran sulfate sodium-induced acute colitis in mice. Saudi J Gastroenterol. (2020) 26:153–9. doi: 10.4103/sjg.SJG_580_19, PMID: 32270772 PMC7392292

[32] Xi M Li J Hao G An X Song Y Wei H. Stachyose increases intestinal barrier through Akkermansia muciniphila and reduces gut inflammation in germ-free mice after human fecal transplantation. Food Res Int. (2020) 137:109288. doi: 10.1016/j.foodres.2020.109288, PMID: 33233042

[33] Bai G Tsuruta T Nishino N. Dietary soy, meat, and fish proteins modulate the effects of prebiotic raffinose on composition and fermentation of gut microbiota in rats. Int J Food Sci Nutr. (2017) 69:480–7. doi: 10.1080/09637486.2017.1382454, PMID: 28958174

[34] Hou Y Wei W Guan X Liu Y Bian G He D. A diet-microbial metabolism feedforward loop modulates intestinal stem cell renewal in the stressed gut. Nat Commun. (2021) 12:271. doi: 10.1038/s41467-020-20673-4, PMID: 33431867 PMC7801547

[35] Stadnicka K Bogucka J Stanek M Graczyk R Krajewski K Maiorano G. Injection of raffinose family oligosaccharides at 12 days of egg incubation modulates the gut development and resistance to opportunistic pathogens in broiler chickens. Animals. (2020) 10:592. doi: 10.3390/ani10040592, PMID: 32244432 PMC7222726

[36] Nagura T Hachimura S Hashiguchi M Ueda Y Kanno T Kikuchi H. Suppressive effect of dietary raffinose on T-helper 2 cell-mediated immunity. Br J Nutr. (2007) 88:421–6. doi: 10.1079/bjn2002666, PMID: 12323091

[37] Dai Z Su D Zhang Y Sun Y Hu B Ye H. Immunomodulatory activity *in vitro* and *in vivo* of verbascose from mung beans (Phaseolus aureus). J Agric Food Chem. (2014) 62:10727–35. doi: 10.1021/jf503510h, PMID: 25317918

[38] Li T Lu X Yang X. Stachyose-enriched α-galacto-oligosaccharides regulate gut microbiota and relieve constipation in mice. J Agric Food Chem. (2013) 61:11825–31. doi: 10.1021/jf404160e, PMID: 24245736

[39] Malech, Harry L. “The role of neutrophils in the immune system: an overview.” Methods in molecular biology. (Clifton, N.J.) vol. 412 (2007): 3–11. doi: 10.1007/978-1-59745-467-4_1, PMID: 18453101

[40] Mercier FE Ragu C Scadden DT. The bone marrow at the crossroads of blood and immunity. Nat Rev Immunol. (2011) 12:49–60. doi: 10.1038/nri3132, PMID: 22193770 PMC4013788

[41] Epstein RS Basu Roy UK Aapro M Salimi T Moran D Krenitsky J. Cancer patients’ Perspectives and experiences of chemotherapy-induced myelosuppression and its impact on daily life. Patient Preference Adherence. (2021) 15:453–65. doi: 10.2147/ppa.S292462, PMID: 33658769 PMC7920579

[42] Zhao S Peng X Zhou Q-Y Huang Y-Y Rao X Tu J-L. Bacillus coagulans 13002 and fructo-oligosaccharides improve the immunity of mice with immunosuppression induced by cyclophosphamide through modulating intestinal-derived and fecal microbiota. Food Res Int. (2021) 140:109793. doi: 10.1016/j.foodres.2020.109793, PMID: 33648160

[43] Sun J Yao N Lu P Wang Y. Effects of mFOLFOX6 regimen combined with carrelizumab on immune function and prognosis in patients with microsatellite instability colorectal cancer. Cell Mol Biol. (2022) 67:356–62. doi: 10.14715/cmb/2021.67.5.48, PMID: 35818232

[44] Thaiss CA Zmora N Levy M Elinav E. The microbiome and innate immunity. Nature. (2016) 535:65–74. doi: 10.1038/nature18847, PMID: 27383981

[45] Shi N Li N Duan X Niu H. Interaction between the gut microbiome and mucosal immune system. Military Med Res. (2017) 4:14. doi: 10.1186/s40779-017-0122-9, PMID: 28465831 PMC5408367

[46] Lapébie P Lombard V Drula E Terrapon N Henrissat B. Bacteroidetes use thousands of enzyme combinations to break down glycans. Nat Commun. (2019) 10:2043. doi: 10.1038/s41467-019-10068-5, PMID: 31053724 PMC6499787

[47] McKee LS La Rosa SL Westereng B Eijsink VG Pope PB Larsbrink J. Polysaccharide degradation by the Bacteroidetes: mechanisms and nomenclature. Environ Microbiol Rep. (2021) 13:559–81. doi: 10.1111/1758-2229.12980, PMID: 34036727

[48] Cai M Zhu H Xu L Wang J Xu J Li Z. Structure, anti-fatigue activity and regulation on gut microflora *in vivo* of ethanol-fractional polysaccharides from Dendrobium officinale. Int J Biol Macromolecules. (2023) 234:123572. doi: 10.1016/j.ijbiomac.2023.123572, PMID: 36754265

[49] Zhu K Fan H Zeng S Nie S Zhang Y Tan L. Polysaccharide from Artocarpus heterophyllus Lam. (jackfruit) pulp modulates gut microbiota composition and improves short-chain fatty acids production. Food Chem. (2021) 364:130434. doi: 10.1016/j.foodchem.2021.130434, PMID: 34182368

[50] Lau N-S Ting SY Sam K-K M J Wong SC Wu X. Comparative analyses of scylla olivacea gut microbiota composition and function suggest the capacity for polyunsaturated fatty acid biosynthesis. Microbial Ecol. (2022) 86:575–88. doi: 10.1007/s00248-022-02046-0, PMID: 35618944

[51] Bradley E Haran J. The human gut microbiome and aging. Gut Microbes. (2024) 16:2359677. doi: 10.1080/19490976.2024.2359677, PMID: 38831607 PMC11152108

[52] Wastyk HC Fragiadakis GK Perelman D Dahan D Merrill BD Yu FB. Gut-microbiota-targeted diets modulate human immune status. Cell. (2021) 184:4137–53.e4114. doi: 10.1016/j.cell.2021.06.019, PMID: 34256014 PMC9020749

[53] Jang S-E. Lactobacillus plantarum HY7712 ameliorates cyclophosphamide-induced immunosuppression in mice. J Microbiol Biotechnol. (2013) 23:414–21. doi: 10.4014/jmb.1210.10010, PMID: 23462016

[54] Neugebauer ME Hsu A Arbab M Krasnow NA McElroy AN Pandey S. Engineered bacteria increase L-arginine to improve immunotherapy response. Cancer Discov. (2021) 11:2956–6. doi: 10.1158/2159-8290.Cd-rw2021-146, PMID: 34654704

[55] Canale FP Basso C Antonini G Perotti M Li N Sokolovska A. Metabolic modulation of tumours with engineered bacteria for immunotherapy. Nature. (2021) 598:662–6. doi: 10.1038/s41586-021-04003-2, PMID: 34616044

