# Supplementary material for: Oligosaccharides from the seeds of Dolichos lablab L. promote gut microbial metabolite L-arginine production to alleviate cyclophosphamide-induced immunosuppression
Source: Front Immunol. 2025 Jul 16;16:1587426. doi: 10.3389/fimmu.2025.1587426 (PMC12307210; doi:10.3389/fimmu.2025.1587426)
Supplement: Supplementary file 1 [file DataSheet1.docx]

**Supplementary material**

**Oligosaccharides from the seeds of *Dolichos lablab L.* promote gut microbial metabolite L-arginine production to alleviate cyclophosphamide-induced immunosuppression**

Ben Liu^a,b,1^, Jiayi Jin^a,1^, Yuming Zhou^b,1^, Zhipeng Shang^a,e^, Peng Meng^d^, Maoru Du^a^, Feng Geng^a^, Xue Gao^a^, Feng Zhao^a,e,^*, Zhenguo Su^b^*, Xiaohong Pan^a,c^*

^a^ School of Pharmacy, Binzhou Medical University, Yantai264003, China;

^b^ Yantai Affiliated Hospital of Binzhou Medical University, Yantai264003, China;

^c^ Binzhou Medical University, Yantai Hospital of Traditional Chinese Medicine, Yantai264003, China;

^d^ Yantai Hospital of Traditional Chinese Medicine, Binzhou Medical University, Yantai264003, China;

^e^ The Key Laboratory of Prescription Effect and Clinical Evaluation of State Administration of Traditional Chinese Medicine of China, Yantai264003, China.

*Corresponding authors (Xiaohong Pan, email: panxiaohong@bzmc.edu.cn; Zhenguo Su, email: szg68@126.com; Feng Zhao, email: zhaofeng2015@163.com)

^1^ Ben Liu, Jiayi Jin and Yuming Zhou contributed equally to this work.

Supplementary Table 1

^1^H and ^13^C NMR chemical shifts for stachyose (ppm).

| Sugar residue | Unit | H-1/C-1 | H-2/C-2 | H-3/C-3 | H-4/C-4 | H-5/C-5 | H-6/C-6 |
| --- | --- | --- | --- | --- | --- | --- | --- |
| Gal-(1→ | a | 4.91/97.99 | 3.74/68.26 | 3.75/69.45 | 3.90/69.41 | 3.91/70.97 | 3.66/61.12 |
| →6)-Gal-(1→ | b | 4.90/98.34 | 3.74/68.41 | 3.82/69.34 | 3.95/69.31 | 4.06/68.77 | 3.64,3.78/66.45 |
| →6)-Glc-(1→ | c | 5.34/92.08 | 3.48/70.92 | 3.67/72.69 | 3.44/69.48 | 3.97/71.24 | 3.58,3.96/65.82 |
| Fru-(1→ | d | 3.58/61.38 | –/103.77 | 4.13/76.30 | 3.97/73.96 | 3.81/81.32 | 3.69,3.74/62.44 |


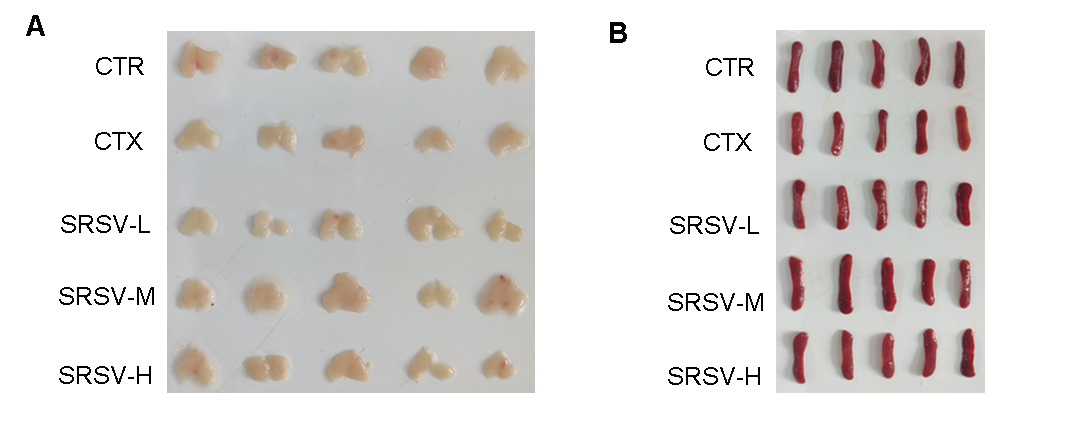


Figure S1 Morphological observation of the thymus and spleen in mice after SRSV intervention. (A) thymus; (B) spleen.


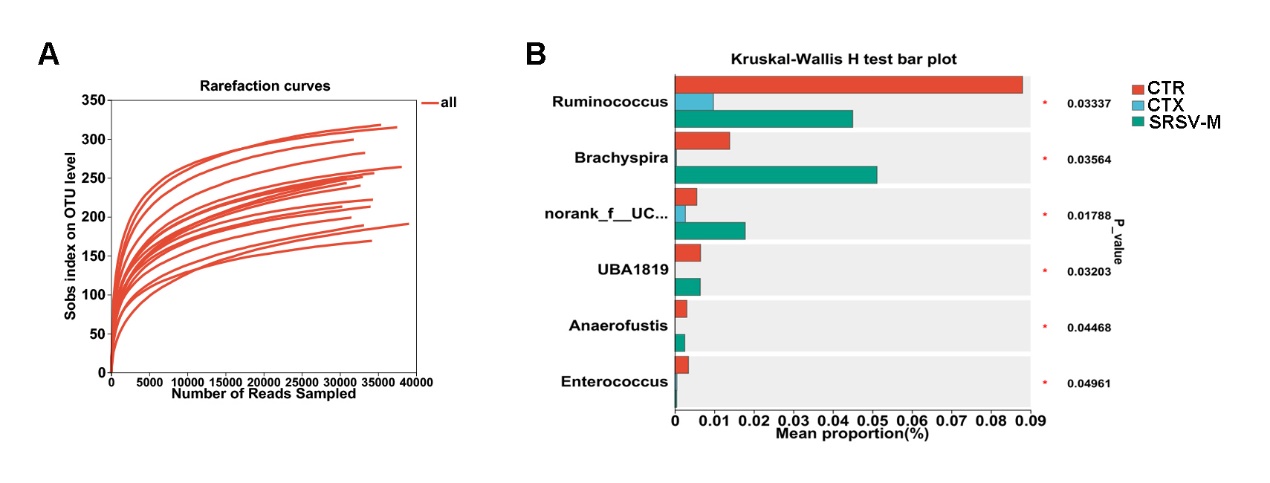


Figure S2 Analysis of intestinal microbiota diversity. (A) Sample dilution curve of the Sobs index. (B) LEfSe analysis of species with significant differences among groups at the taxonomic level from class to genus (LDA > 2).


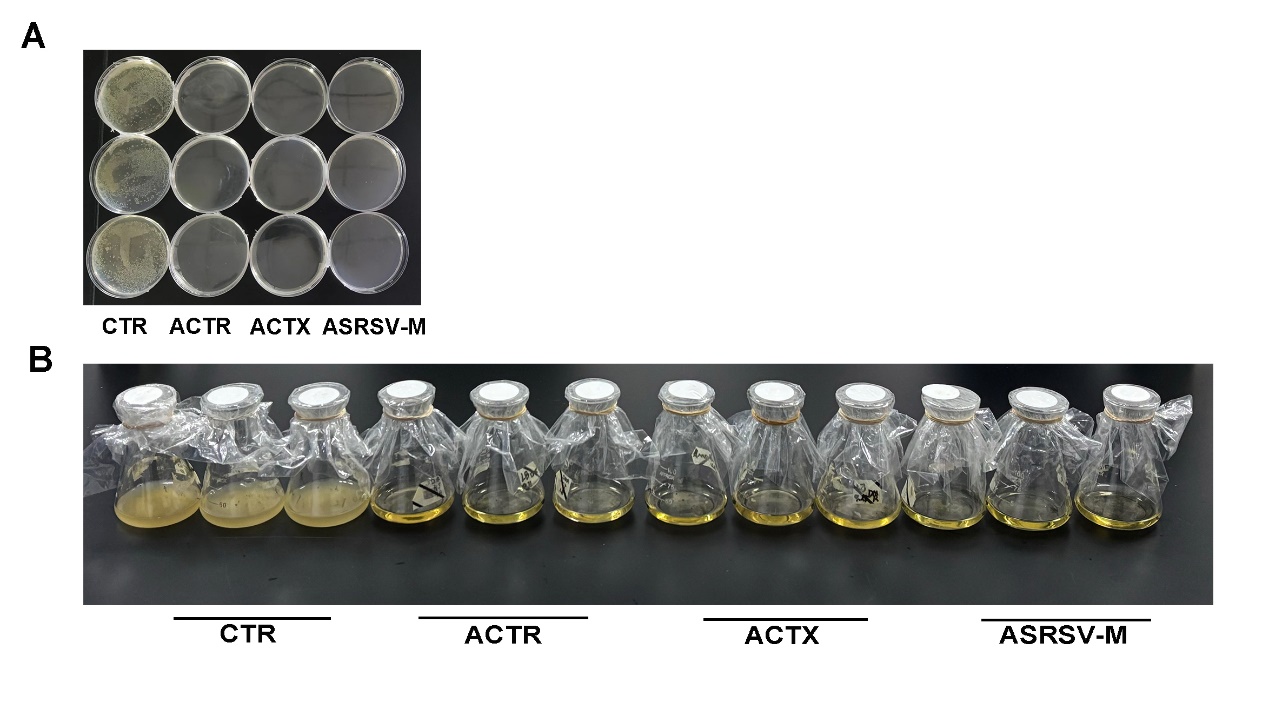


Figure S3 Detection of intestinal bacteria in broad-spectrum antibiotics (ABX)-treated mice. After 7 days of ABX treatment, the colony culture of mouse feces on LB solid medium (A), and the colony culture in LB liquid medium (B). (n=3)


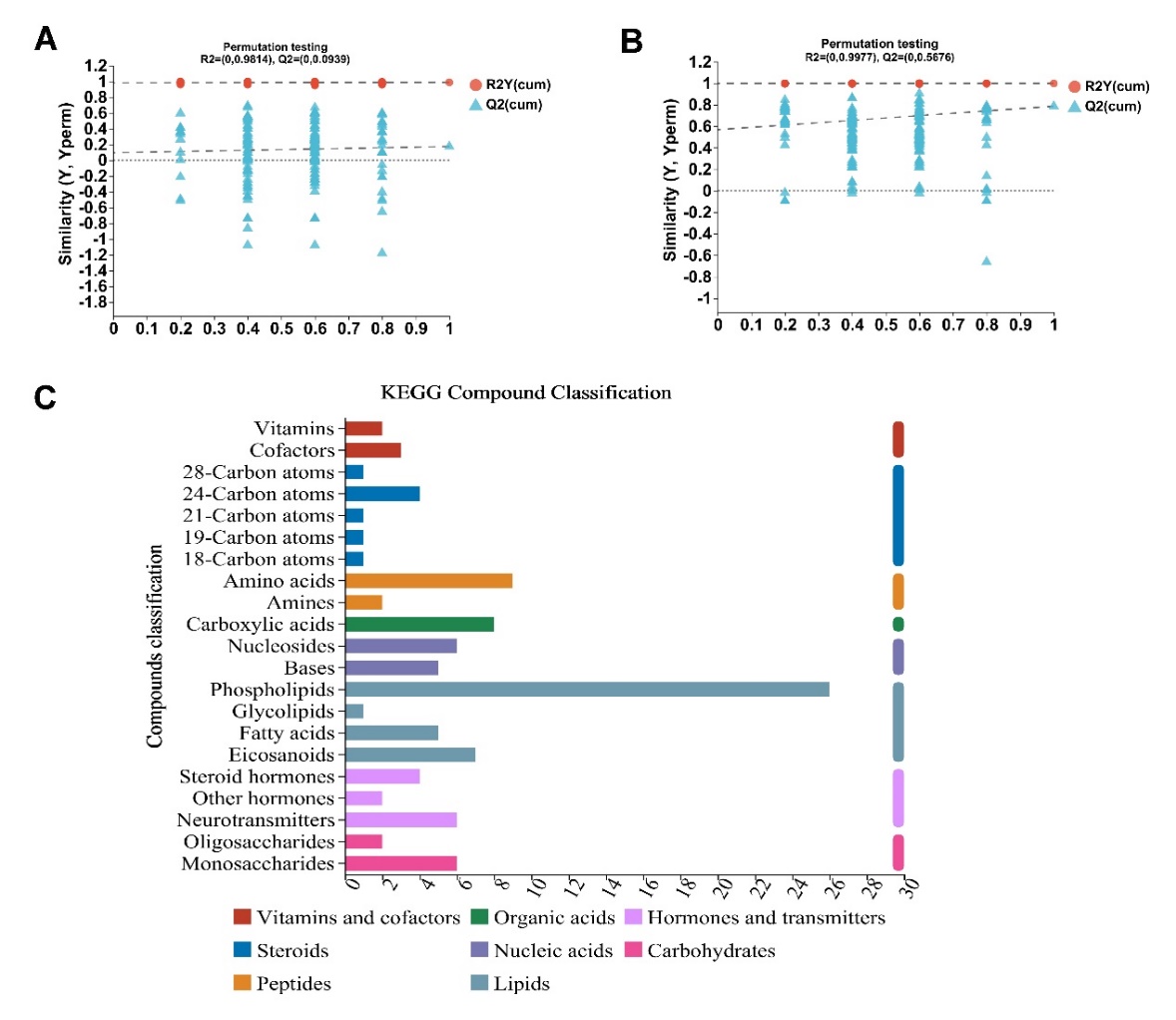


Figure S4 Metabolomic analysis. (A) PLS-DA model validation (cationic model). (B) PLS-DA model validation (anionic mode). (C) KEGG compound composition and the content of each classification of all metabolites detected in the three groups of CTR, CTX and SRSV-M.
